# BST2 confers cisplatin resistance via NF-*κ*B signaling in nasopharyngeal cancer

**DOI:** 10.1038/cddis.2017.271

**Published:** 2017-06-15

**Authors:** Chun-mei Kuang, Xiang Fu, Yi-jun Hua, Wen-di Shuai, Zhi-hua Ye, Yingchang Li, Qi-hua Peng, Yi-zhuo Li, Shuai Chen, Chao-nan Qian, Wenlin Huang, Ran-yi Liu

**Affiliations:** 1 Sun Yat-sen University Cancer Center, State Key Laboratory of Oncology in South China, Collaborative Innovation Center of Cancer Medicine, Guangzhou, China; 2 Department of Radio-chemotherapy, Shangrao People’s Hospital, Shangrao, Jiangxi, China; 3 Department of Nasopharyngeal Carcinoma, Sun Yat-sen University Cancer Center, Guangzhou, China; 4 Guangdong Provincial Key Laboratory of Tumor-targeted Drug & Guangzhou Enterprise Key Laboratory of Gene Medicine, Guangzhou Doublle Bioproducts Co., Ltd., Guangzhou, China

## Abstract

Concurrent/adjuvant cisplatin-based chemoradiotherapy is regarded as the standard of treatment for locoregionally advanced nasopharyngeal carcinoma (NPC). However, patients who do not respond to cisplatin suffer, rather than benefit, from chemotherapy treatment. The goal of this study was to identify molecules involved in cisplatin resistance and to clarify their molecular mechanisms, which would help in the discovery of potential therapeutic targets and in developing a personalized and precise treatment approach for NPC patients. We previously generated a cisplatin-sensitive NPC cell line, S16, from CNE2 cells and found that eIF3a, ASNS and MMP19 are upregulated in S16 cells, which contributes to their cisplatin sensitivity. In this study, we found that BST2 is downregulated in cisplatin-sensitive S16 cells compared with CNE2 cells. Knockdown of BST2 in NPC cells sensitized their response to cisplatin and promoted cisplatin-induced apoptosis, whereas exogenous overexpression of BST2 increased their cisplatin resistance and inhibited cisplatin-induced apoptosis. Further investigation demonstrated that BST2-mediated cisplatin resistance depended on the activation of the NF-*κ*B signaling pathway and consequent upregulation of anti-apoptotic genes, such as Bcl-X_L_ and livin. Moreover, an analysis of clinical data revealed that a high BST2 level might serve as an independent indicator of poor prognosis in patients with locally advanced NPC treated with platinum-based chemoradiotherapy. These findings suggest that BST2 likely mediates platinum resistance in NPC, offering guidance for personalized and precise treatment strategies for patients with NPC.

Nasopharyngeal carcinoma (NPC) is a highly prevalent malignancy in South-East Asia and Africa but is rare in Western countries. Moreover, the incidence rates of this disease have been increasing in recent years, especially in South-East China, East Asia and North Africa.^1, 2^ Based on the National Comprehensive Cancer Network (NCCN) guidelines (Version 2.2016), concurrent/adjuvant platinum-based chemoradiotherapy has become the standard treatment for locoregionally advanced NPC.^3, 4, 5^ Specifically, the addition of chemotherapy to radiotherapy significantly improves overall survival compared with radiotherapy alone,^6^ though concurrent chemoradiotherapy is associated with a higher incidence of severe late toxicity in NPC patients. In other words, many patients unfortunately benefit less and suffer more from concurrent chemoradiotherapy.^7^ Because treatment failure is likely due to intrinsic or acquired platinum resistance by tumors,^8^ clarifying the molecular mechanisms of platinum resistance in NPC is important for developing personalized and precise platinum-based therapeutic approaches.^9^

BST2, also known as Tetherin, CD317 or HM1.24, is a well-known cellular restriction factor that blocks release of HIV-1, Kaposi's sarcoma-associated herpesvirus, Ebola virus, and other enveloped viruses from the cell surface.^10, 11, 12^ However, BST2 was first identified as a type II transmembrane glycoprotein expressed in bone marrow stromal cell lines and involved in pre-B-cell growth.^13^ Accumulating evidence has shown that BST2 is also constitutively expressed on various other immune cells and malignant cells,^14, 15^ such as breast cancer,^16^ oral cavity cancer,^17^ lung cancer,^18^ B-cell chronic lymphocytic leukemia^19^ and gastrointestinal cancer cells.^20^ Nonetheless, BST2 is not elevated in all cancers. For example, BST2 levels are significantly downregulated in lung squamous cell carcinoma, kidney papillary cell and chromophobe carcinoma, liver cancer, prostate cancer and B-cell acute lymphoblastic leukemia, and BST2 levels are unchanged in lung adenocarcinoma and thyroid cancer.^15, 19^ Thus, the role of BST2 in NPC remains unclear.

In previous studies, we generated the cisplatin-sensitive subclone S16 from CNE2 cells^21^ and found that, as evidenced by mRNA microarray data, BST2 is downregulated in S16 cells compared with the cisplatin-resistant parental CNE2 cells.^22^ In the present study, BST2 was identified as a platinum-resistant factor in NPC. Upregulation of BST2 resulted in platinum resistance by activating the NF-*κ*B pathway to promote the expression of anti-apoptotic proteins. Moreover, BST2 upregulation was associated with poor survival in patients with locally advanced NPC treated with platinum-based chemoradiation.

## Results

### BST2 contributes to cisplatin resistance in CNE2 cells

In a previous study, we reported that ASNS and MMP19, which are upregulated in cisplatin-sensitive S16 cells compared with in their parental CNE2 cells, conferred cisplatin sensitivity to NPC.^22^ Here, we focused on genes that are downregulated in S16 cells based on our previous analysis.^22^ By combining a literature review and qPCR analysis, BST2, MVK, FGF2 and FOXK2, which exhibited downregulated expression in S16 cells compared with in the parental CNE2 cells either before or after cisplatin treatment (Supplementary Figure 1a), were selected as candidates for investigating relationships with platinum resistance. A primary MTT (3-(4,5-dimethylthiazol-2-yl)-2,5-diphenyltetrazolium bromide) assay revealed that the half-maximal inhibitory concentration (IC_50_) of cisplatin was reduced to some extent by knockdown the expression of BST2, MVK, FGF2 and FOXK2 genes in CNE2 cells. As this effect was most pronounced for BST2 gene (Supplementary Figure 1b), we selected this target for further investigation.

We then evaluated BST2 expression in CNE2 and S16 cells by western blotting (WB), and found that the BST2 protein level was also lower in S16 cells than in CNE2 cells (Supplementary Figure 1c). Furthermore, cisplatin treatment did not affect BST2 expression (Supplementary Figure 1d). Thus, we speculated that BST2 plays an essential role in the cisplatin resistance of CNE2 cells and that BST2 may be an intrinsic, not acquired, factor that mediates cisplatin resistance.

**Figure 1:**
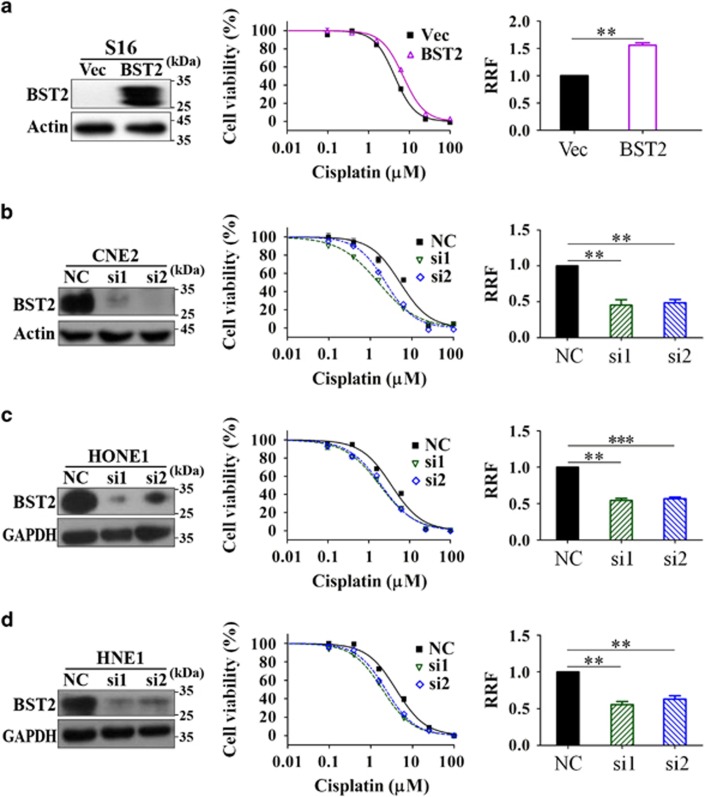
Contributions of BST2 to cisplatin resistance in nasopharyngeal cancer (NPC) cells. (**a**) S16 cells stably overexpressing BST2 or (**b**) CNE2, (**c**) HONE1 and (**d**) HNE1 cells transfected with BST2 siRNAs for 24 h were subjected to MTT assays, as described in Materials and Methods (Vec: empty vector transfected; BST2: BST2 overexpression; si1, si2, NC: BST2 siRNAs or negative control siRNA transfected). Left: western blotting (WB) analysis of BST2 expression at 48 h after siRNA transfection; actin or GAPDH was used as a loading control. Middle: representative dose-dependent cell viability curves. Right: averages of relative resistant factors (RRFs, the ratios of IC_50_ value of experimental cells to that of control cells) in 3–5 independent experiments (**P<0.01; ***P<0.001)

To assess the contribution of BST2 to cisplatin resistance, we first overexpressed BST2 in S16 cells via stable transfection of lentiviruses containing BST2 cDNA and then measured the IC_50_ of cisplatin. The results showed that the IC_50_ of cisplatin was increased in S16 cells stably overexpressing BST2, and the relative resistance factor (RRF) increased by 55.0% in these cells compared with that in the vector-transfected control cells (*P*<0.01; Figure 1a). These data indicate that upregulation of BST2 increases cisplatin resistance.

As confirmation, we next knocked down BST2 expression in CNE2 cells via transient transfection of BST2 siRNAs and investigated the effect on cisplatin resistance. The results showed that two siRNAs successfully knocked down BST2 expression in CNE2 cells, which effectively reduced the cisplatin IC_50_ (Figure 1b). Specifically, the RRF to cisplatin decreased by 56.0% (si1) and 43.3% (si2) in these cells compared with control CNE2 cells (*P*<0.01; Figure 1b). Thus, high BST2 expression likely contributes to the cisplatin resistance of CNE2 cells compared with their cisplatin-sensitive subclone, S16 cells. These results suggest that BST2 overexpression contributes to the cisplatin resistance in NPC CNE2 cell line.

### The contribution of BST2 to the cisplatin resistance is not cell line specific

To assess whether the contribution of BST2 to cisplatin resistance is specific to the CNE2 cell line and its derivative cell line, we next investigated the effect of BST2 on cisplatin resistance in another two NPC cell lines, HONE1 and HNE1, by knocking down BST2 expression with siRNAs and determining the IC_50_ of cisplatin by an MTT assay. The results showed that reduced BST2 expression sensitized both cell lines to cisplatin and decreased the IC_50_ values (Figures 1c and d). Compared with the respective control cells transfected with scrambled siRNA, the RRFs decreased by 46% (si1) and 41.6% (si2) in HONE1 cells and by 43.9% (si1) and 37.5% (si2) in HNE1 cells (Figures 1c and d; all *P*<0.01). For the three NPC cell lines, the RRFs of cells transfected with BST2-si1 were lower than those of cells transfected with BST2-si2 (Figures 1b–d), but the differences are very small and not significant. Thus, we conclude that BST2 contributes to cisplatin resistance in NPC cells and that this effect is not specific to the CNE2 cell line.

### BST2 suppresses cisplatin-induced apoptosis in NPC cells

**Figure 2:**
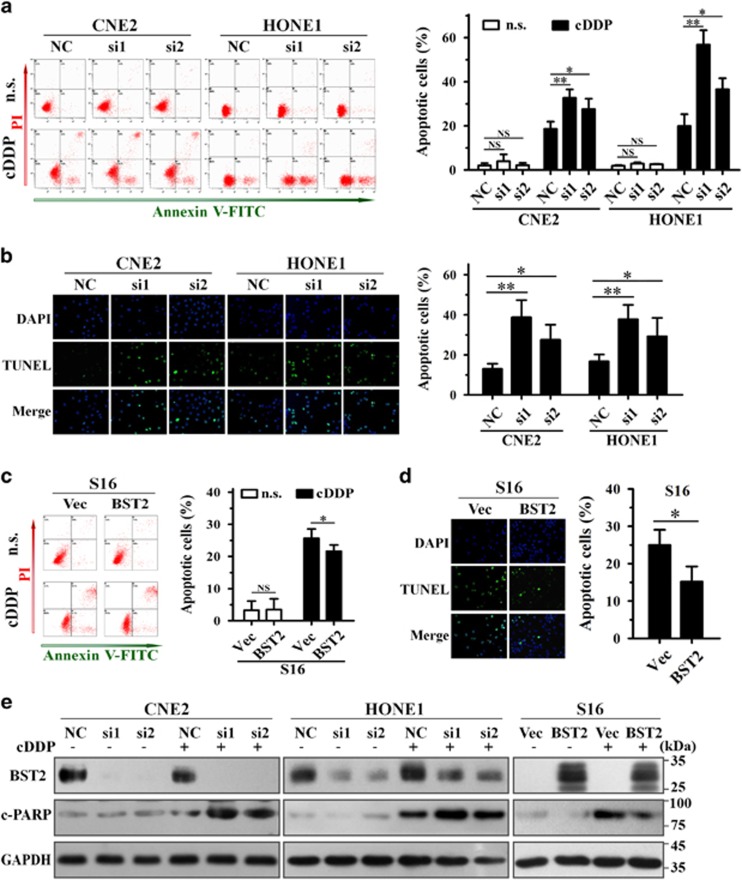
Effect of BST2 on cisplatin-induced apoptosis. NPC cells transiently transfected with siRNAs for 24 h or cells stably overexpressing BST2 were treated with cisplatin (cDDP) for 24 h, and apoptosis was analyzed. The experiments were independently repeated for 3–4 times. (**a**,**c**) Annexin-V-FITC/PI dual staining assay (left, representative plots for flow cytometry; right, bar charts indicating the average percentages of apoptotic cells). (**b**,**d**) TUNEL assays (left, representative immunofluorescent pictures; right, bar charts indicating the average percentages of apoptotic cells). (**e**) WB assays (c-PARP, cleaved PARP; GAPDH, a loading control). NC, negative control siRNA; si1, si2, BST2 siRNAs; Vec, empty vector transfected; BST2, BST2 overexpression; n.s., normal saline. *P<0.05; **P<0.01; NS, no significance

Because cisplatin exerts its anticancer effect by forming platinum-DNA adducts, resulting in DNA damage and consequent apoptosis, we hypothesized that BST2 inhibited cisplatin-induced apoptosis in NPC cells. Annexin-V/propidium iodide (PI) staining revealed that silencing BST2 expression significantly enhanced cisplatin-induced (*P*<0.05 or <0.01) but not spontaneous apoptosis in CNE2 and HONE1 cells (Figure 2a). Furthermore, terminal deoxynucleotidyl transferase (TdT) dUTP nick-end labeling (TUNEL) assays showed similar results: BST2 knockdown sensitized NPC cells to cisplatin-induced apoptosis (*P*<0.05 or <0.01; Figure 2b). In contrast, S16 cells stably overexpressing BST2 were more resistant to cisplatin-induced apoptosis; BST2 overexpression reduced the number of Annexin-V-positive S16 cells after cisplatin treatment (*P*<0.05; Figure 2c) and decreased the number of TUNEL-positive cells (*P*<0.05; Figure 2d) compared with control cells.

Because poly (ADP-ribose) polymerase (PARP) cleavage is a common marker of apoptosis, we also investigated the effect of BST2 on cisplatin-induced PARP cleavage in NPC cells. The results showed that BST2 knockdown increased PARP cleavage in CNE2 and HONE1 cells treated with cisplatin, whereas BST2 overexpression reduced PARP cleavage in cisplatin-treated S16 cells, compared with the respective control cells (Figure 2e). Thus, BST2 contributes to cisplatin resistance and suppression of cisplatin-induced apoptosis in NPC cells.

### BST2 promotes the expression of anti-apoptotic genes in NPC cells

**Figure 3:**
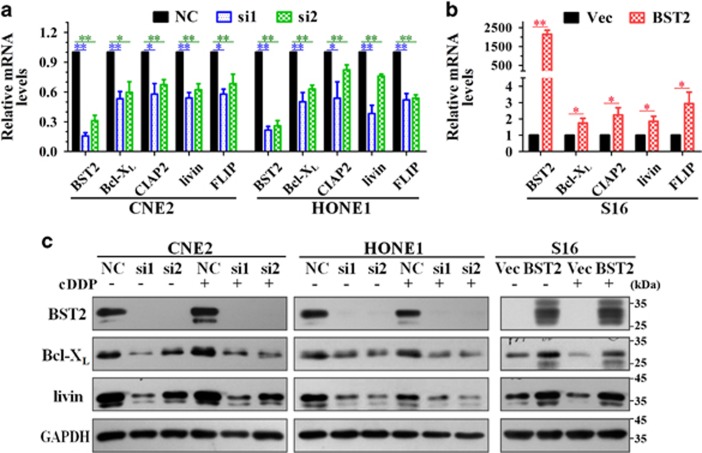
BST2 upregulates expression of anti-apoptotic factors in NPC cells. After 48 h of transfection with BST2 siRNAs in CNE2 and HONE1 cells or with BST2 cDNA in S16 cells, qPCR and WB assays were performed to measure effects of BST2 on the expression of anti-apoptotic genes (NC, negative control siRNA; si1, si2, BST2 siRNAs; Vec, empty vector; BST2, BST2 cDNA). (**a**) Relative mRNA levels of anti-apoptotic factors in CNE2 and HONE1 cells. (**b**) Relative mRNA levels of anti-apoptotic factors in S16 cells (*P<0.05, **P<0.01). (**c**) WB assay for Bcl-X_L_ and livin proteins in NPC cells (GAPDH was used as a loading control)

We then speculated that BST2 inhibits cisplatin-induced apoptosis by promoting the expression of anti-apoptotic genes. Thus, we examined the effects of BST2 on the expression of anti-apoptotic genes, such as Bcl-X_L_, CIAP2, livin and FLIP.^23, 24, 25, 26^ According to our results, BST2 knockdown was accompanied by decreases in mRNA levels of Bcl-X_L_, CIAP2, livin and FLIP in CNE2 and HONE1 cells (Figure 3a), whereas ectopic overexpression of BST2 increased transcription of these genes in S16 cells (Figure 3b). Similar results were observed for Bcl-X_L_ and livin at the protein levels in the presence and absence of cisplatin (Figure 3c). These data suggest that BST2 inhibits cisplatin-induced apoptosis in NPC cells by promoting the expression of anti-apoptotic genes.

### The activation of the NF-*κ*B pathway is likely responsible for BST2-induced cisplatin resistance in NPC cells

**Figure 4:**
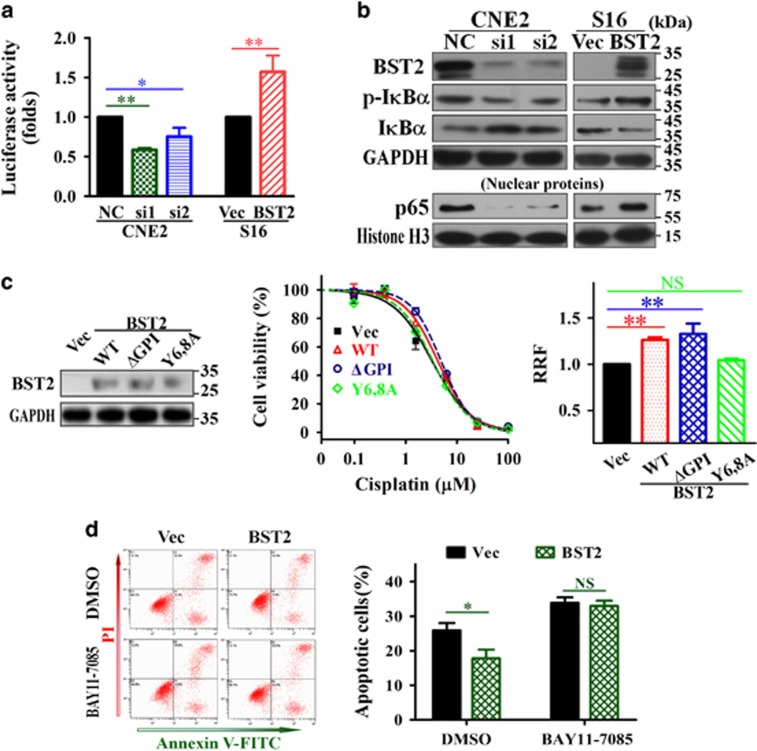
Activation of NF-*κ*B signaling by BST2 is required for cisplatin resistance in NPC cells. (**a**) NF-*κ*B activity assay. NPC cells were transfected with BST2 siRNAs (si1, si2) or cDNA (BST2) for 24 h followed by a luciferase reporter assay, as described in Materials and Methods. (**b**) WB assays for total I*κ*B*α*, phosphor-I*κ*B*α* (p-I*κ*B*α*) and nuclear p65 levels (GAPDH and histone H3 were used as loading controls) in CNE2 cells transfected BST2 siRNAs or S16 cells transfected with BST2 cDNA for 48 h and treated with 10 *μ*M cisplatin for 24 h. (**c**) Effects of WT BST2 and BST2 mutants (ΔGPI and Y6,8A) on cisplatin resistance were investigated by MTT assays in S16 cells stably overexpressing WT BST2 or BST2 mutants (left, WB assay for BST2 expression; middle, representative dose-dependent cell viability curves; right, relative resistant factors). (**d**) inhibition of I*κ*B*α* phosphorylation reverses BST2-induced anti-apoptosis. After 24 h of treatment with 10 *μ*M cisplatin plus 10 *μ*M BAY11-7085 (DMSO was used as a vehicle control), apoptosis was analyzed by Annexin-V/PI dual staining (left, representative dot plots for flow cytometry; right, the percentages of apoptotic cells) in S16 cells stably expressing BST2. NC, negative control siRNA; si1, si2, BST2 siRNAs; Vec, empty vector-tranfected; BST2, BST2 overexpression;. *P<0.05, **P<0.01; NS, no significance

To clarify the molecular mechanisms by which BST2 contributes to cisplatin resistance and anti-apoptosis, we first performed a dual luciferase reporter assay in 293T cells to screen for possible signaling pathways involved. As shown in Supplementary Figure 2a, BST2 activated the NF-*κ*B, COX2 and IFN-*β* signaling pathways in 293T cells, and this effect was most pronounced for the NF-*κ*B pathway. BST2 has also previously been reported to restrict HIV infection by activating the NF-*κ*B pathway,^27^ and activation of the NF-*κ*B pathway has been shown to attenuate apoptosis by upregulating the expression of anti-apoptotic factors.^23, 24, 25, 26, 28, 29^ Therefore, we hypothesized that BST2 confers cisplatin resistance by activating NF-*κ*B signaling. As expected, BST2 knockdown attenuated NF-*κ*B pathway activity in HeLa cells with a high level of BST2 (Supplementary Figures 2b and c), and further analysis demonstrated that overexpressing BST2 in S16 cells activated the NF-*κ*B pathway, whereas knocking down BST2 expression inhibited the pathway in CNE2 cells (Figure 4a).

As confirmation of the results of our luciferase reporter assay, NPC cells in which BST2 was knocked down or overexpressed were exposed to cisplatin, and the protein levels of crucial molecules involved in the NF-*κ*B pathway were detected by WB assay. The results demonstrated that, compared with the respective control cells, total I*κ*B*α* was upregulated, p-I*κ*B*α* and nuclear p65 were reduced in CNE2 cells with BST2 knockdown, whereas, total I*κ*B*α* decreased, p-I*κ*B*α* and nuclear p65 increased in BST2-overexpressing S16 cells (Figure 4b). Thus, we conclude that BST2 activates the NF-*κ*B pathway in NPC cells.

To verify the role of NF-*κ*B activation in BST2-induced cisplatin resistance, we first assessed NF-*κ*B signaling activity in 293T and S16 cells overexpressing wild-type BST2 or two types of BST2 mutants, ΔGPI and Y6,8 A. The results showed that NF-*κ*B activity was increased in cells harboring wild-type BST2 and the ΔGPI mutant (*P*<0.01) but almost entirely absent in cells overexpressing the Y6,8 A mutant (Supplementary Figures 2d and e). We then stably overexpressed wild-type BST2 or the BST2 mutants in S16 cells and examined the resulting cisplatin IC_50_ values. The results demonstrated that overexpression of wild-type BST2 and the ΔGPI mutant enhanced cisplatin resistance, as evidence by increases in IC_50_ and RRF values compared with the control (*P*<0.01), whereas overexpression of the Y6,8 A mutant did not affect the cisplatin IC_50_ or RRF (Figure 4c).

Moreover, we also found that suppressing the NF-*κ*B pathway using a small molecular inhibitor of I*κ*B*α* phosphorylation, BAY11-7085 (Supplementary Figure 2f), reversed the effect of BST2 on cisplatin-induced apoptosis (Figure 4d). Based on the above data, we conclude that BST2 enhances cisplatin resistance by activating the NF-*κ*B pathway in NPC cells.

To investigate relationships among BST2, the NF-*κ*B pathway and anti-apoptotic factors downstream of NF-*κ*B, we examined the protein levels of BST2, I*κ*B*α* and Bcl-X_L_ in tumor tissues from patients with NPC by immunohistochemistry (IHC) assay. BST2 expression was found to negatively correlate with I*κ*B*α* expression and positively correlate with Bcl-X_L_ expression; in addition, I*κ*B*α* expression negatively correlated with Bcl-X_L_ expression (Supplementary Figure 3). These findings suggest that BST2 activates NF-*κ*B signaling and then upregulates expression of anti-apoptotic genes downstream of NF-*κ*B to induce platinum resistance in NPC.

### BST2 knockdown reverses the cisplatin resistance of NPC cells by inhibiting NF-*κ*B signaling and consequently downregulating anti-apoptotic factors in xenograft tumors

**Figure 5:**
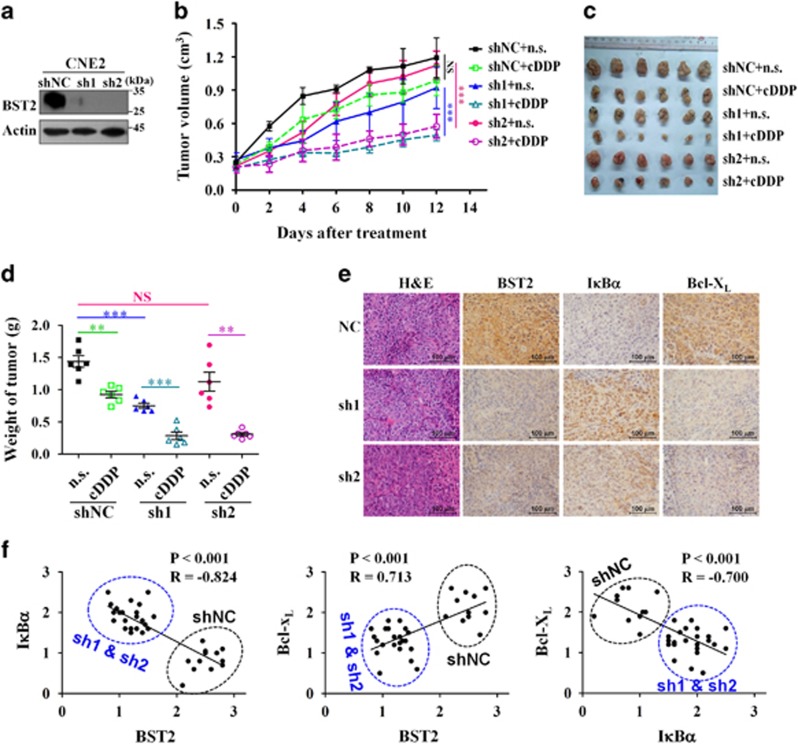
BST2 knockdown reverses cisplatin resistance of NPC cells in xenograft tumors in nude mice. CNE2 cells stably expressing two specific BST2 shRNAs (sh1, sh2) or scrambled control shRNA (shNC) were subcutaneously injected to generate xenograft tumors in nude mice; cDDP treatment was performed as described in Materials and Methods. Normal saline (n.s.) was used as the treatment control. (**a**) Western blot analysis for BST2 expression in stable cell lines. (**b**) Growth curves of tumor xenografts. (**c**) Images of xenograft tumors harvested at the end of the experiment. (**d**) The weights of tumors are presented as a Cleveland dot plot, and the average±S.D. is included (*n*=6/group; **P<0.01; ***P<0.001; NS, no significance). (**e**) Representative IHC images assesing BST2, Bcl-X_L_ and I*κ*B*α* levels in xenograft tissue samples. (**f**) Scatter diagrams for the correlation analysis of BST2, Bcl-X_L_ and I*κ*B*α* expressions (the sources of the points are indicated by broken circles)

We next sought to determine whether BST2 knockdown sensitizes tumors to cisplatin *in vivo* by stably knocking down expression of BST2 in CNE2 cells (CNE2-sh1 and CNE2-sh2; CNE2-shNC as negative control cells) and establishing subcutaneous xenografts in nude mice, which were then treated with cisplatin. The results showed that transfection of BST2 shRNAs effectively decreased BST2 protein levels in CNE2 cells (Figure 5a) and inhibited the growth of NPC xenografts in nude mice. The inhibitory effect of BST2-sh1 was stronger than that of BST2-sh2 (Figures 5b–d), which may be due to the lower level of residual BST2 expression after BST2-sh1 transfection (Figure 5e). Above all, BST2 knockdown enhanced the sensitivity of xenograft tumors to cisplatin (Figures 5b–d), consistent with the results obtained *in vitro*. Specifically, cisplatin treatment inhibited tumor growth by 62.2% in CNE2-sh1 xenografts and 72.7% in CNE2-sh2 xenografts, whereas the CNE2-shNC control group only exhibited 35.7% growth inhibition.

To verify relationships among BST2, the NF-*κ*B pathway and anti-apoptotic factors downstream of NF-*κ*B, the levels of BST2, I*κ*B*α* and Bcl-X_L_ in the xenografts were examined by IHC assay. Similar to our *in vitro* results, I*κ*B*α* was upregulated and Bcl-X_L_ was downregulated in xenografts in which BST2 was stably knocked down compared with the control xenografts (Figures 5e and f). Specifically, BST2, I*κ*B*α* and Bcl-X_L_ expression correlated linearly (Figure 5f), as also observed in clinical NPC tissue samples (Supplementary Figure 3). Taken together, these data indicate that BST2 mediates platinum resistance in NPC and that BST2 inhibition can reverse cisplatin resistance by retarding the NF-*κ*B pathway *in vivo*.

### BST2 upregulation is associated with poor prognosis in patients with locally advanced NPC treated with platinum-based chemoradiotherapy

**Figure 6:**
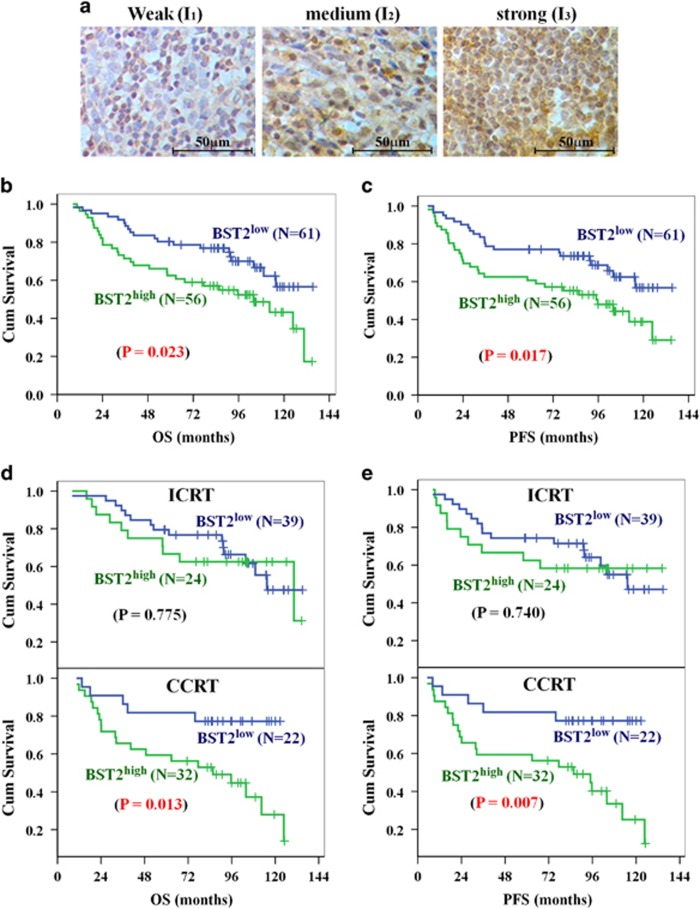
High expression of BST2 predicts poor prognosis in NPC patients treated with platinum-based chemoradiotherapy. (**a**) Representative IHC pictures. Brown staining, BST2 positive; blue, cell nucleus. (**b**,**c**) Kaplan–Meier analysis and the log-rank test for overall survival (OS) (**b**) and progression-free survival (PFS) (**c**) according to BST2 expression level. (**d**,**e**) Stratified analysis for OS (**d**) and PFS (**e**) by treatment strategy. ICRT, induction chemotherapy plus radiotherapy; CCRT, including concurrent chemoradiotherapy only or concurrent chemoradiotherapy plus induction chemotherapy. *P*<0.05 indicates a significant difference (in red)

As BST2 is associated with platinum resistance in NPC cell lines, we assessed the prognostic value of BST2 in patients with NPC treated with platinum-based chemotherapy. To this end, we randomly selected 117 patients who were newly diagnosed with locally advanced NPC and treated with radiotherapy plus cisplatin-based chemotherapy (at least 2 cycles) (Supplementary Table 1), and examined BST2 expression by IHC in biopsy tumor specimens. The results showed that BST2 was expressed in all samples and highly expressed (H score ≥1.85) in 47.9% of samples (Supplementary Table 1). A two-tailed *χ*^2^ test revealed that BST2 expression did not significantly correlate with clinicopathologic parameters, including gender, age (<50, ≥50), T classification (T_1-2_, T_3-4_), N classification (N_0-1_, N_2-3_), clinical stage and histological type (Supplementary Table 2). Multivariable Cox regression analysis showed that T classification and BST2 expression can serve as independent prognostic factors for overall survival (OS) (BST2, *P*=0.015, HR 2.10, 95% CI 1.16–3.82) and progression-free survival (PFS) (BST2, *P*=0.011, HR 2.13, 95% CI 1.19–3.82) (Table 1). Due to the small sample size, N classification was not included as an independent prognostic factor in this study, though it did tend to correlate with OS (*P*=0.085) and PFS (*P*=0.084). In addition, Kaplan–Meier survival analysis suggested that high BST2 expression can predict poor prognosis in patients with locally advanced NPC treated with platinum-based chemoradiotherapy, as evidenced by shorter OS (*P*=0.023, log-rank test; Figure 6b) and PFS (*P*=0.017, log-rank test; Figure 6c).

We also performed a subgroup survival analysis to evaluate the prognostic value of BST2 expression (OS and PFS) in patient subgroups stratified by treatment strategy. The results demonstrated that BST2 expression better predicted OS and PFS in the subgroups treated only with concurrent chemoradiotherapy (CCRT) or with CCRT plus induction chemotherapy (IC) than in the subgroup treated with induction chemotherapy plus radiotherapy (ICRT) (Figures 6d and e). Additionally, subgroup survival analysis stratified by cisplatin treatment cycles showed that high BST2 expression can predict poor prognosis only in patients receiving radiation and more than 3 cycles of cisplatin-based chemotherapy, whereas the BST2 level lacked predictive value in patients receiving fewer cycles of chemotherapy (Supplementary Figures 4a, b and Supplementary Table 3). These findings suggest that the poor prognosis associated with a high BST2 level is likely caused not by radiation resistance but by platinum resistance.

## Discussion

Concurrent/adjuvant cisplatin-based chemoradiotherapy is a common treatment strategy for locoregionally advanced NPC according to NCCN guidelines.^3, 4, 5, 6^ However, patients who do not respond to cisplatin treatment suffer, rather than benefit, from chemotherapy.^7^ Thus, identifying molecular markers involved in cisplatin resistance is very important for developing personalized and precise treatments for NPC patients.^9^ In previous studies, we found that eIF3a, ASNS and MMP19 inhibited expression of nucleotide excision repair (NER) proteins and/or anti-apoptotic factors, consequently conferring cispaltin sensitivity to NPC cells.^21, 22^ In the present study, we show that BST2/Tetherin promotes platinum resistance by activating the NF-*κ*B pathway to induce expression of anti-apoptotic proteins in NPC cells. Moreover, upregulated BST2 was found to be associated with poor survival in patients with locally advanced NPC treated with platinum-based chemoradiation (Supplementary Figure 5).

Cisplatin and other platinum-based antitumor drugs have been used to treat a wide range of tumors. The primary anticancer mechanism of platinum is to bind covalently to DNA and form platinum-DNA adducts, which results in apoptosis,^30^ and apoptosis dysregulation is one of the primary mechanisms underlying cisplatin resistance.^22, 31, 32^ In this paper, we reported that BST2 inhibited cisplatin-induced apoptosis in NPC cells and that this effect may be associated with upregulation of anti-apoptotic factors. Specifically, knockdown of BST2 downregulated the anti-apoptotic factors (such as Bcl-X_L_ and livin), whereas overexpression of BST2 upregulated these factors in NPC cells. Moreover, the levels of Bcl-X_L_ protein positively correlated with BST2 in the cancer tissues of NPC patients. These findings support the notion that BST2 confers cisplatin resistance by upregulating anti-apoptotic gene expression.

The NF-*κ*B pathway is a well-known survival-related pathway that upregulates multiple anti-apoptotic genes, and its activation has been found to increase chemoresistance and tumor progression.^33, 34^ Many anti-apoptotic genes, including Bcl-X_L_, CIAP2 and livin, have been identified as downstream target genes regulated by NF-*κ*B signaling.^23, 24, 25^ In this study, BST2 was shown to activate the NF-*κ*B pathway in NPC cells, consistent with previous reports in 293, 293T and HeLa cells.^14, 27, 35^ Indeed, the innate immune or inflammatory responses triggered by activation of NF-*κ*B pathway, together with the viral particle-tethering effect, make BST2 a powerful virus restriction factor.^15^ According to our data, the YXY motif, but not the GPI anchor, of BST2 is necessary for its signaling function, as deletion of GPI could not attenuate BST2-mediated NF-*κ*B activation; in constrast, the Y6,8A mutation almost completely abrogated NF-*κ*B activation in 293T and S16 cells. These findings are also supported by the report of Guatelli J and colleagues^27^ but somewhat contradict those of Galao RP *et al.*^35^ Overexpression of the BST2 Y6,8A mutant did not increase platinum resistance in NPC cells, whereas overexpressing the ΔGPI mutant and wild-type BST2 had similar effects on platinum resistance, corroborating the effects of these constructs on NF-*κ*B activation. Additionally, BST2 protein levels positively correlated with Bcl-X_L_ levels and negatively correlated with I*κ*B*α* levels in xenografts in nude mice and cancer tissues of NPC patients. These findings confirm that the impact of BST2 on platinum resistance depends on activation of NF-*κ*B signaling and the consequent upregulation of anti-apoptotic factors. We also found that BST2 can activate COX2 signaling in 293T cells, though the contribution of COX2 signaling to BST2-directed cisplatin resistance warrants further study.

BST2 is upregulated in several malignancies,^15^ and high levels of BST2 are associated with poor prognosis in patients with oral cavity, esophageal, gastric and colorectal cancers.^17, 36, 37^ BST2 is also reported to promote the growth and metastasis of breast cancer.^16, 38^ Thus, BST2 may have a proto-oncogenic role and promotes cancer progression,^38^ which is also consistant with our observation that knockdown of BST2 delayed the growth of NPC xenografts in nude mice.

Our retrospective clinical investigation demonstrated that high levels of BST2 in cancer tissues may serve as an independent poor prognostic factor for patients with locally advanced NPC treated with platinum-based chemoradiotherapy. However, the poor prognosis of patients expressing high levels of BST2 is likely due to platinum resistance, and not to increased NPC cell proliferation or radiation resistance, because BST2 expression level was only associated with prognosis in patients who received >3 cycles of platinum-based chemotherapy and not in patients who received fewer cycles when they received similar doses of radiation. Similarly, high BST2 expression was associated with shorter survival in patients receiving CCRT (include CCRT+IC) but not in patients receiving ICRT, which may result from differences in the cycles of platinum-based chemotherapy between the CCRT group and ICRT group (4.38 cycles *versus* 2.22 cycles).

NPC is an Epstein-Barr virus (EBV)-associated malignant tumor, and all non-keratinizing cases of NPC involve EBV infection. These tumors can be divided into two subtypes, World Health Organization (WHO) types II and III. Type III NPC is the most common subtype, and a class of interferon-stimulated genes (ISGs), including BST2, are significantly activated in type III tumors compared with type II tumors. Consistent with this finding, endogenous EBV replication is suppressed in type III NPC, rendering the virus latent.^39^ Thus, we speculate that elevated BST2 expression likely results from latent EBV infection in type III NPC, and that BST2 may have multiple functions in EBV latency, oncogenesis and cisplatin resisitance. However, we did not find differences in BST2 levels between type III and type II NPC, which may be due to the insufficient number of type II samples in this study.

Overall, BST2 is downregulated in cisplatin-sensitive NPC cells and upregulated in cisplatin-resistant NPC cells. BST2 overexpression results in platinum resistance in NPC, which depends on activation of the NF-*κ*B pathway and consequent upregulation of anti-apoptotic factors. Moreover, BST2 upregulation is associated with poor prognosis in patients with locally advanced NPC treated with platinum-based chemoradiotherapy (Supplementary Figure 5). These findings indicate that BST2 mediates platinum resistance in NPC. Thus, BST2 may serve as a predictive biomarker in the development of personalized and precise treatment strategies and as a treatment target to reverse chemotherapy resistance in future clinical practice.

## Materials and methods

### Cells, siRNAs, plasmids and transient transfection

The human NPC cell lines HONE1, HNE1,^40^ CNE2 (ref. 41) and CNE2-derived cisplatin-sensetive clone S16,^21^ human cervical cancer HeLa cells and human embryonic kidney 293T cells were cultured in Dulbecco's modified Eagle medium (DMEM) containing 10% fetal bovine serum (Gibco, Grand Island, NY, USA) at 37 °C in 5% CO_2_ and saturated humidity. Negative control small-interfering RNA (NC) and siRNAs targeting human BST2 (Supplementary Table 4) were synthesized by GenePharma (Suzhou, Jiangsu, China). A plasmid expressing BST2 was constructed by inserting the human BST2 cDNA, cloned by reverse transcription-PCR with specific primers (Supplementary Table 5), into the pcDNA3.1 vector. Furthermore, pcDNA3.1 plasmids expressing BST2/ΔGPI (an in-frame deletion GPI anchor-defective mutant of BST2, Δ156–162) and BST2/Y6,8A (in which the tyrosines at positions 6 and 8 are replaced with alanines) were gifts from Dr John Guatelli of the University of California-San Diego.^27^ For transient transfection, plasmids or siRNAs were transfected using Lipofectamine 2000 (Invitrogen, Carlsbad, CA, USA) according to the manufacturer’s instructions. After 24 h of incubation, cells were harvested by trypsinization for further analysis.

### Establishment of stable cell lines

Wild-type (WT) BST2 cDNA or BST2 mutants were amplified by PCR using the above plasmids as templates (primers are listed in Supplementary Table 5) and subcloned into the PCDH-EF1-MCS-T2A-Puro plasmid to construct lentiviral expression vectors expressing WT BST2 or BST2 mutants. To construct the lentiviral BST2 shRNA expression vector, synthesized double-strand oligodeoxynucleotides with sticky ends (Supplementary Table 4) were inserted into the Age I and EcoR I sites of the pLKO.1 plasmid. The lentiviruses were packaged by co-transfecting expression plasmids and a lentiviral packaging kit (FulenGen, Guangzhou, China) into 293T cells. The virus supernatant was collected at 48 h after transfection and then used to infect target cells in the presence of 8 *μ*g/ml of polybrene (Sigma-Aldrich, St Louis, MO, USA). Stable cell lines were selected following treatment with 2 *μ*g/ml puromycin (Sigma-Aldrich) for 2 weeks.

### RNA extraction, reverse transcription and quantitative real-time PCR

Total cellular RNAs were extracted using TRIzol reagent (Invitrogen) and transcribed with M-MLV Reverse Transcriptase (Promega, Madison, WI, USA). The mRNA levels were measured by quantitative real-time PCR (qPCR) using a SYBR Green PCR Kit (Bio-Rad Laboratories, Hercules, CA, USA) with specific primers (Supplementary Table 5). GAPDH was used as an internal control. Threshold cycle (Ct) values were normalized against that of the GAPDH internal control.^42^ Relative mRNA levels are presented as the 2^−ΔCt^ value normalized to the control group.

### Western blotting assay

Western blotting was performed as previously described.^43, 44^ Briefly, the cells were collected and lysed using lysis buffer containing protease inhibitor (Sigma-Aldrich) on ice for 30 min followed by centrifugation. Protein concentrations in the supernatants were determined using a protein assay kit (Bio-Rad), and samples were separated by 8–12% sodium dodecyl sulfate-polyacrylamide gel electrophoresis and transferred to polyvinylidene fluoride membranes. After blocking for nonspecific binding, the blots were incubated with specific antibodies against BST2 (Abcam, Cambridge, MA, USA), cleaved PARP (c-PARP), Bcl-X_L_, livin (Cell Signaling Technology, Beverly, MA, USA), I*κ*B*α*, phospho-I*κ*B*α*, actin (Santa Cruz Biotechnology, Santa Cruz, CA, USA) or GAPDH (Proteintech Group, Chicago, IL, USA), followed by reaction with an HRP-conjugated secondary antibody (Cell Signaling Technology). Signals were enhanced with the ECL detection system (Amersham Biosciences, Piscataway, NJ, USA) and captured using X-ray film.

### Cell viability assay

Cell viability was measured using an MTT assay, as previously described.^45, 46^ Briefly, NPC cells transiently transfected with siRNAs or stably overexpressing BST2 were seeded in 96-well plates (2000 cells/well) and incubated for 24 h before being exposed to different concentrations of cisplatin for 72 h. The cells were stained with MTT (Invitrogen), and the OD_490nm_ was measured. The data were analyzed using GraphPad Prism 5 (GraphPad Software, La Jolla, CA, USA) to obtain the IC_50_. The relative resistance factor (RRF) was calculated by dividing the IC_50_ value by that of the control group.

### Cell apoptosis analysis

Apoptosis was analyzed with Annexin-V/PI binding and TUNEL assays. For the Annexin-V/PI binding assay,^47^ NPC cells transiently transfected with siRNAs or stably overexpressing BST2 were exposed to 10 *μ*M cisplatin with or without 10 *μ*M BAY11-7085 (Selleck, Houston, TX, USA) for 24 h; floating and adherent cells were then collected by centrifugation and trypsinization respectively. The cells were then resuspended in binding buffer and subsequently stained with FITC-Annexin-V and PI (BestBio, Shanghai, China). Stained cells were quantified using a FACScan flow cytometer (BD Biosciences, Franklin lakes, NJ, USA) according to the manufacturer’s directions.

For the TUNEL assay,^48^ NPC cells transiently transfected with siRNAs or stably overexpressing BST2 were incubated with cisplatin for 24 h in glass-bottom cell culture dishes (NEST Biotechnology, Wuxi, China). The cells were then subjected to a TUNEL assay using DeadEnd Fluorometric TUNEL System (Promega). After counterstaining with 4',6-diamidino-2-phenylindole (DAPI) (Invitrogen), the cells were immediately examined under a fluorescence microscope.

### Luciferase reporter assay

A luciferase reporter assay was employed to analyze the activity of signaling pathway.^49, 50^ Briefly, cells in 24-well plates were co-transfected with 500 ng of luciferase reporter plasmids consisting of a promoter containing the indicated transcription factor binding sequence or response element (Addgene, Cambridge, MA, USA) and 10 ng of internal control pRL-TK Renilla plasmid (Promega). After 24 h, the cells were lysed, and the luciferase and Renilla signals were measured using Dual Luciferase Reporter Assay Kit (Promega) according to the manufacturer’s instructions.

### Animal experiments

All animal experiments were conducted according to current Chinese regulations and standards regarding the use of laboratory animals, and all experiments were approved by the Sun Yat-sen University Cancer Center Institutional Animal Care and Usage Committee. Female BALB/c nude mice (4–5 weeks old, 18–20 g) were purchased from SLRC laboratory animals (Shanghai, China). The mice were randomly divided into 3 groups (12/group) and subcutaneously injected with CNE2 cells stably expressing BST2 shRNA1, shRNA2 or scramble shRNA (1 × 10^6^ in 100 *μ*l PBS/mouse). Xenograft development was monitored every 2 days. When the tumors reached a volume of approximately 200 mm^3^, the mice in each group were randomly divided into 2 subgroups and treated with intraperitoneal injections of cisplatin (3 mg/kg) or an equal volume (100 *μ*l) of normal saline (NS) every 2 days for approximately 2 weeks. The mice were sacrificed, and the tumors were removed and weighed. The tumor volume was evaluated using the following formula: tumor volume=4*π*/3 × (width/2)^2^ × (length/2).^49^

### Patient tissue specimens and clinicopathological characteristics

Paraffin-embedded tissues were collected from 117 patients with locally advanced NPC without distant metastasis NPC and treated with platinum-based chemotherapy at the Sun Yat-sen University Cancer Center (SYSUCC) between 2002 and 2009. All patients received platinum-based chemotherapy (at least two cycles) combined with radiotherapy (68–72Gy for the nasopharynx and 60–66Gy for lymph nodes). The study protocol was approved by the institutional review board and human ethics committee of SYSUCC, and informed consent was obtained from each patient. The baseline characteristics of the NPC patients are shown in Supplementary Table 1.

### Immunohistochemical analysis

Immunohistochemistry (IHC) was performed as previously described.^51, 52^ Briefly, paraffin sections were deparaffinized, rehydrated, blocked with hydrogen peroxide and subjected to antigen-retrieval by heating for 25 min in citrate buffer (pH8.0). The slides were then incubated with primary antibodies at 4 °C overnight, followed by incubation with an HRP-conjugated secondary antibody and visualization with peroxidase Envision Kit (Dako, Carpinteria, CA, USA); hematoxylin was used for counterstaining. Two pathologists evaluated the slides independently in a double-blinded manner. Protein levels were assessed by the H score, which was evaluated based on a proportion score (P_1-3_) and an intensity score (I_0_, I_1-3_) and dichotomized according to OS by a receiver operating characteristic (ROC) curve. Representative IHC images of BST2 (for I_1_–I_3_) are shown in Figure 6a.

### Statistical analysis

All *in vitro* experiments were repeated at least three times, and the animal experiments were repeated twice. The data were analyzed with SPSS 16.0 (SPSS, Chicago, IL, USA). *P*<0.05 was considered to indicate a significant difference.

## Figures and Tables

**Table 1:** Multivariate Cox regression analysis of prognostic factors for NPC patients

**Variable**	**OS**		**PFS**	
	**HR (95% CI)**	***P*-value**	**HR (95% CI)**	***P*-value**
Gender (male *versus* female)	0.801 (0.431–1.488)	0.483	0.739 (0.403–1.356)	0.329
Age (≥50 *versus* <50)	1.386 (0.764–2.517)	0.283	1.389 (0.777–2.482)	0.268
T classification (T_3-4_ *versus* T_1-2_)	3.453 (1.395–8.548)	0.007**	3.106 (1.277–7.555)	0.012*
N classification (N_2-3_ *versus* N_0-1_)	1.688 (0.930–3.064)	0.085	1.667 (0.933–2.978)	0.084
Treatment strategy (CCRT^a^ *versus* ICRT^b^)	1.269 (0.704–2.288)	0.428	1.089 (0.610–1.942)	0.773
BST2 expression (high *versus* low)	2.100 (1.156–3.817)	0.015*	2.131 (1.191–3.815)	0.011*

Abbreviations: CI, confidence interval; HR, hazard ratio; OS, overall survival; PFS, progression-free survival

a CCRT, including concurrent chemoradiotherapy (CCRT) induction chemotherapy plus CCRT

b ICRT, induction chemotherapy plus radiotherapy (RT).* or **, significant difference

## References

[bib1] Tang LL, Chen WQ, Xue WQ, He YQ, Zheng RS, Zeng YX et al. Global trends in incidence and mortality of nasopharyngeal carcinoma. Cancer Lett 2016; 374: 22–30. 26828135 10.1016/j.canlet.2016.01.040

[bib2] Chen W, Zheng R, Zeng H, Zhang S. The incidence and mortality of major cancers in China, 2012. Chin J Cancer 2016; 35: 73. 27484217 10.1186/s40880-016-0137-8 PMC4971631

[bib3] Qiu WZ, Huang PY, Shi JL, Xia HQ, Zhao C, Cao KJ. Neoadjuvant chemotherapy plus intensity-modulated radiotherapy versus concurrent chemoradiotherapy plus adjuvant chemotherapy for the treatment of locoregionally advanced nasopharyngeal carcinoma: a retrospective controlled study. Chin J Cancer 2016; 35: 2. 26739148 10.1186/s40880-015-0076-9 PMC4704429

[bib4] Chen L, Hu CS, Chen XZ, Hu GQ, Cheng ZB, Sun Y et al. Concurrent chemoradiotherapy plus adjuvant chemotherapy versus concurrent chemoradiotherapy alone in patients with locoregionally advanced nasopharyngeal carcinoma: a phase 3 multicentre randomised controlled trial. Lancet Oncol 2012; 13: 163–171. 22154591 10.1016/S1470-2045(11)70320-5

[bib5] Guan Y, Liu S, Wang HY, Guo Y, Xiao WW, Chen CY et al. Long-term outcomes of a phase II randomized controlled trial comparing intensity-modulated radiotherapy with or without weekly cisplatin for the treatment of locally recurrent nasopharyngeal carcinoma. Chin J Cancer 2016; 35: 20. 26879049 10.1186/s40880-016-0081-7 PMC4753647

[bib6] Chan AT, Leung SF, Ngan RK, Teo PM, Lau WH, Kwan WH et al. Overall survival after concurrent cisplatin-radiotherapy compared with radiotherapy alone in locoregionally advanced nasopharyngeal carcinoma. J Natl Cancer Inst 2005; 97: 536–539. 15812080 10.1093/jnci/dji084

[bib7] Du CR, Ying HM, Kong FF, Zhai RP, Hu CS. Concurrent chemoradiotherapy was associated with a higher severe late toxicity rate in nasopharyngeal carcinoma patients compared with radiotherapy alone: a meta-analysis based on randomized controlled trials. Radiat Oncol 2015; 10: 70. 25889937 10.1186/s13014-015-0377-9 PMC4464879

[bib8] Galluzzi L, Senovilla L, Vitale I, Michels J, Martins I, Kepp O et al. Molecular mechanisms of cisplatin resistance. Oncogene 2012; 31: 1869–1883. 21892204 10.1038/onc.2011.384

[bib9] Amable L. Cisplatin resistance and opportunities for precision medicine. Pharmacol Res 2016; 106: 27–36. 26804248 10.1016/j.phrs.2016.01.001

[bib10] Neil SJ, Zang T, Bieniasz PD. Tetherin inhibits retrovirus release and is antagonized by HIV-1 Vpu. Nature 2008; 451: 425–430. 18200009 10.1038/nature06553

[bib11] Douglas JL, Gustin JK, Viswanathan K, Mansouri M, Moses AV, Fruh K. The great escape: viral strategies to counter BST-2/tetherin. PLoS Pathog 2010; 6: e1000913. 20485522 10.1371/journal.ppat.1000913 PMC2869331

[bib12] Simon V, Bloch N, Landau NR. Intrinsic host restrictions to HIV-1 and mechanisms of viral escape. Nat Immunol 2015; 16: 546–553. 25988886 10.1038/ni.3156 PMC6908429

[bib13] Ishikawa J, Kaisho T, Tomizawa H, Lee BO, Kobune Y, Inazawa J et al. Molecular cloning and chromosomal mapping of a bone marrow stromal cell surface gene, BST2, that may be involved in pre-B-cell growth. Genomics 1995; 26: 527–534. 7607676 10.1016/0888-7543(95)80171-h

[bib14] Cocka LJ, Bates P. Identification of alternatively translated Tetherin isoforms with differing antiviral and signaling activities. PLoS Pathog 2012; 8: e1002931. 23028328 10.1371/journal.ppat.1002931 PMC3460627

[bib15] Mahauad-Fernandez WD, Okeoma CM. The role of BST-2/Tetherin in host protection and disease manifestation. Immunity Inflamm Dis 2016; 4: 4–23. 10.1002/iid3.92 PMC4768070 27042298

[bib16] Mahauad-Fernandez WD, DeMali KA, Olivier AK, Okeoma CM. Bone marrow stromal antigen 2 expressed in cancer cells promotes mammary tumor growth and metastasis. Breast Cancer Res 2014; 16: 493. 25499888 10.1186/s13058-014-0493-8 PMC4308845

[bib17] Fang KH, Kao HK, Chi LM, Liang Y, Liu SC, Hseuh C et al. Overexpression of BST2 is associated with nodal metastasis and poorer prognosis in oral cavity cancer. Laryngoscope 2014; 124: E354–E360. 24706327 10.1002/lary.24700

[bib18] Wang W, Nishioka Y, Ozaki S, Jalili A, Abe S, Kakiuchi S et al. HM1.24 (CD317) is a novel target against lung cancer for immunotherapy using anti-HM1.24 antibody. Cancer Immunol Immunother 2009; 58: 967–976. 18979097 10.1007/s00262-008-0612-4 PMC11030068

[bib19] Gong S, Osei ES, Kaplan D, Chen YH, Meyerson H. CD317 is over-expressed in B-cell chronic lymphocytic leukemia, but not B-cell acute lymphoblastic leukemia. Int J Clin Exp Pathol 2015; 8: 1613–1621. 25973046 PMC4396245

[bib20] Mukai S, Oue N, Oshima T, Mukai R, Tatsumoto Y, Sakamoto N et al. Overexpression of transmembrane protein BST2 is associated with poor survivalof patients with esophageal, gastric, or colorectal cancer. Ann Surg Oncol 2016; 24: 594–602. 26832883 10.1245/s10434-016-5100-z

[bib21] Liu RY, Dong Z, Liu J, Yin JY, Zhou L, Wu X et al. Role of eIF3a in regulating cisplatin sensitivity and in translational control of nucleotide excision repair of nasopharyngeal carcinoma. Oncogene 2011; 30: 4814–4823. 21625209 10.1038/onc.2011.189 PMC3165083

[bib22] Liu RY, Dong Z, Liu J, Zhou L, Huang W, Khoo SK et al. Overexpression of asparagine synthetase and matrix metalloproteinase 19 confers cisplatin sensitivity in nasopharyngeal carcinoma cells. Mol Cancer Ther 2013; 12: 2157–2166. 23956056 10.1158/1535-7163.MCT-12-1190 PMC3795908

[bib23] Wang CY, Mayo MW, Korneluk RG, Goeddel DV, Baldwin AS Jr. NF-kappaB antiapoptosis: induction of TRAF1 and TRAF2 and c-IAP1 and c-IAP2 to suppress caspase-8 activation. Science 1998; 281: 1680–1683. 9733516 10.1126/science.281.5383.1680

[bib24] Deveraux QL, Reed JC. IAP family proteins—suppressors of apoptosis. Genes Dev 1999; 13: 239–252. 9990849 10.1101/gad.13.3.239

[bib25] Chen C, Edelstein LC, Gelinas C. The Rel/NF-kappaB family directly activates expression of the apoptosis inhibitor Bcl-x(L). Mol Cell Biol 2000; 20: 2687–2695. 10733571 10.1128/mcb.20.8.2687-2695.2000 PMC85484

[bib26] Ding L, Chen S, Liu P, Pan Y, Zhong J, Regan KM et al. CBP loss cooperates with PTEN haploinsufficiency to drive prostate cancer: implications for epigenetic therapy. Cancer Res 2014; 74: 2050–2061. 24491799 10.1158/0008-5472.CAN-13-1659 PMC3975662

[bib27] Tokarev A, Suarez M, Kwan W, Fitzpatrick K, Singh R, Guatelli J. Stimulation of NF-kappaB activity by the HIV restriction factor BST2. J Virol 2013; 87: 2046–2057. 23221546 10.1128/JVI.02272-12 PMC3571454

[bib28] Li ZH, Tang QB, Wang J, Zhou L, Huang WL, Liu RY et al. Hepatitis C virus core protein induces malignant transformation of biliary epithelial cells by activating nuclear factor-kappaB pathway. J Gastroenterol Hepatol 2010; 25: 1315–1320. 20594262 10.1111/j.1440-1746.2009.06201.x

[bib29] Tan L, Jia H, Liu R, Wu J, Han H, Zuo Y et al. Inhibition of NF-kappaB in fusogenic membrane glycoprotein causing HL-60 cell death: implications for acute myeloid leukemia. Cancer Lett 2009; 273: 114–121. 18783878 10.1016/j.canlet.2008.07.035

[bib30] Wang D, Lippard SJ. Cellular processing of platinum anticancer drugs. Nat Rev Drug Discov 2005; 4: 307–320. 15789122 10.1038/nrd1691

[bib31] Ahmad S. Platinum-DNA interactions and subsequent cellular processes controlling sensitivity to anticancer platinum complexes. Chem Biodivers 2010; 7: 543–566. 20232326 10.1002/cbdv.200800340

[bib32] Kong LR, Chua KN, Sim WJ, Ng HC, Bi C, Ho J et al. MEK inhibition overcomes cisplatin resistance conferred by SOS/MAPK pathway activation in squamous cell carcinoma. Mol Cancer Ther 2015; 14: 1750–1760. 25939760 10.1158/1535-7163.MCT-15-0062

[bib33] Sun Y, Guan Z, Liang L, Cheng Y, Zhou J, Li J et al. NF-kappaB signaling plays irreplaceable roles in cisplatin-induced bladder cancer chemoresistance and tumor progression. Int J Oncol 2016; 48: 225–234. 26647959 10.3892/ijo.2015.3256

[bib34] Tanaka K, Babic I, Nathanson D, Akhavan D, Guo D, Gini B et al. Oncogenic EGFR signaling activates an mTORC2-NF-kappaB pathway that promotes chemotherapy resistance. Cancer Discov 2011; 1: 524–538. 22145100 10.1158/2159-8290.CD-11-0124 PMC3229221

[bib35] Galao RP, Le Tortorec A, Pickering S, Kueck T, Neil SJ. Innate sensing of HIV-1 assembly by Tetherin induces NFkappaB-dependent proinflammatory responses. Cell Host Microbe 2012; 12: 633–644. 23159053 10.1016/j.chom.2012.10.007 PMC3556742

[bib36] Mukai S, Oue N, Oshima T, Mukai R, Tatsumoto Y, Sakamoto N et al. Overexpression of transmembrane protein BST2 is associated with poor survival of patients with esophageal, gastric, or colorectal cancer. Ann Surg Oncol 2017; 24: 594–602. 26832883 10.1245/s10434-016-5100-z

[bib37] Chiang SF, Kan CY, Hsiao YC, Tang R, Hsieh LL, Chiang JM et al. Bone marrow stromal antigen 2 is a novel plasma biomarker and prognosticator for colorectal carcinoma: a secretome-based verification study. Dis Markers 2015; 2015: 874054. 26494939 10.1155/2015/874054 PMC4606116

[bib38] Cai D, Cao J, Li Z, Zheng X, Yao Y, Li W et al. Up-regulation of bone marrow stromal protein 2 (BST2) in breast cancer with bone metastasis. BMC Cancer 2009; 9: 102. 19338666 10.1186/1471-2407-9-102 PMC2674058

[bib39] Pegtel DM, Subramanian A, Meritt D, Tsai CH, Sheen TS, Golub TR et al. IFN-alpha-stimulated genes and Epstein-Barr virus gene expression distinguish WHO type II and III nasopharyngeal carcinomas. Cancer Res 2007; 67: 474–481. 17234754 10.1158/0008-5472.CAN-06-1882

[bib40] Glaser R, Zhang HY, Yao KT, Zhu HC, Wang FX, Li GY et al. Two epithelial tumor cell lines (HNE-1 and HONE-1) latently infected with Epstein-Barr virus that were derived from nasopharyngeal carcinomas. Proc Natl Acad Sci USA 1989; 86: 9524–9528. 2556716 10.1073/pnas.86.23.9524 PMC298529

[bib41] Han H, Zhong C, Zhang X, Liu R, Pan M, Tan L et al. Genistein induces growth inhibition and G2/M arrest in nasopharyngeal carcinoma cells. Nutr Cancer 2010; 62: 641–647. 20574925 10.1080/01635581003605490

[bib42] Qiu L, Wu J, Pan C, Tan X, Lin J, Liu R et al. Downregulation of CDC27 inhibits the proliferation of colorectal cancer cells via the accumulation of p21Cip1/Waf1. Cell Death Dis 2016; 7: e2074. 26821069 10.1038/cddis.2015.402 PMC4816181

[bib43] Du WY, Lu ZH, Ye W, Fu X, Zhou Y, Kuang CM et al. The loss-of-function mutations and down-regulated expression of ASB3 gene promote the growth and metastasis of colorectal cancer cells. Chin J Cancer 2017; 36: 11. 28088228 10.1186/s40880-017-0180-0 PMC5237493

[bib44] Yin JY, Dong ZZ, Liu RY, Chen J, Liu ZQ, Zhang JT. Translational regulation of RPA2 via internal ribosomal entry site and by eIF3a. Carcinogenesis 2013; 34: 1224–1231. 23393223 10.1093/carcin/bgt052 PMC3670257

[bib45] Fu X, Hu J, Han HY, Hua YJ, Zhou L, Shuai WD et al. High expression of XPA confers poor prognosis for nasopharyngeal carcinoma patients treated with platinum-based chemoradiotherapy. Oncotarget 2015; 6: 28478–28490. 26156020 10.18632/oncotarget.4424 PMC4695073

[bib46] Liu J, Wu J, Zhou L, Pan C, Zhou Y, Du W et al. ZD6474, a new treatment strategy for human osteosarcoma, and its potential synergistic effect with celecoxib. Oncotarget 2015; 6: 21341–21352. 26050198 10.18632/oncotarget.4179 PMC4673269

[bib47] Liu RY, Zhu YH, Zhou L, Zhao P, Li HL, Zhu LC et al. Adenovirus-mediated delivery of interferon-gamma gene inhibits the growth of nasopharyngeal carcinoma. J Transl Med 2012; 10: 256. 23272637 10.1186/1479-5876-10-256 PMC3573957

[bib48] Zhao P, Zhu YH, Wu JX, Liu RY, Zhu XY, Xiao X et al. Adenovirus-mediated delivery of human IFNgamma gene inhibits prostate cancer growth. Life Sci 2007; 81: 695–701. 17714738 10.1016/j.lfs.2007.05.028

[bib49] Shi W, Ye Z, Zhuang L, Li Y, Shuai W, Zuo Z et al. Olfactomedin 1 negatively regulates NF-kappaB signalling and suppresses the growth and metastasis of colorectal cancer cells. J Pathol 2016; 240: 352–365. 27555280 10.1002/path.4784

[bib50] Chen S, Sheng C, Liu D, Yao C, Gao S, Song L et al. Enhancer of zeste homolog 2 is a negative regulator of mitochondria-mediated innate immune responses. J Immunol 2013; 191: 2614–2623. 23918984 10.4049/jimmunol.1203143

[bib51] Zhou Y, Wu J, Fu X, Du W, Zhou L, Meng X et al. OTUB1 promotes metastasis and serves as a marker of poor prognosis in colorectal cancer. Mol Cancer 2014; 13: 258. 25431208 10.1186/1476-4598-13-258 PMC4351937

[bib52] Ye W, Liu R, Pan C, Jiang W, Zhang L, Guan Z et al. Multicenter randomized phase 2 clinical trial of a recombinant human endostatin adenovirus in patients with advanced head and neck carcinoma. Mol Ther 2014; 22: 1221–1229. 24662947 10.1038/mt.2014.53 PMC4048902

