# Supplementary material for: BST2 confers cisplatin resistance via NF-κB signaling in nasopharyngeal cancer
Source: Cell Death Dis. 2017 Jun 15;8(6):e2874–. doi: 10.1038/cddis.2017.271 (PMC5520926; doi:10.1038/cddis.2017.271)
Supplement: Supplementary Tables [file cddis2017271x1.doc]

**Supplemental Information**

**Supplemental Tables**

**Table S1. Baseline characteristics of NPC patients.**

| **Variable** |  | **No. of patients (%)** |
| --- | --- | --- |
| Gender | Male  Female | 88 (75.2)  29 (24.8) |
| Age | < 50  ≥ 50 | 72 (61.5)  45 (38.5) |
| T classification | T_1_  T_2_  T_3_  T_4_ | 3 ( 2.6)  23 (19.7)  63 (53.8)  28 (23.9) |
| N classification | N_0_  N_1_  N_2_  N_3_ | 13 (11.1)  44 (37.6)  56 (47.9)  4 ( 3.4) |
| Clinical stage | II  III  IVa/b | 5 ( 4.3)  81 (69.2)  31 (26.5) |
| Histological type | WHO type II  WHO type III | 5 ( 4.3)  112 (95.7) |
| EBV VCA/IgA | Negative  Positive | 2 ( 1.7)  113 (98.3) |
| Treatment strateg | IC^a^ + RT^b^  CCRT^c^ +IC^a^  CCRT^c^ only | 63 (53.8)  34 (29.1)  20 (17.1) |
| Survival status | Alive  Death | 67 (57.3)  50 (42.7) |
| BST2 levels | Low (H score < 1.85)  High (H score ≥ 1.85) | 61 (52.1)  56 (47.9) |

Note: ^a^. IC, induction chemotherapy. ^b^. RT, radiotherapy. ^c^. CCRT, concurrent chemoradiotherapy.

**Table S2. Correlation between BST2 levels and clinicopathologic features.**

| **Characteristic** | **BST2^low^ (%)** | **BST2^high^ (%)** | **P value** |
| --- | --- | --- | --- |
| Gender  Male  Female | 45 (73.8)  16 (26.2) | 43 (76.8)  13 (23.2) | 0.706 ^a^ |
| Age (Medium age-yr)  < 50  ≥ 50 | 47.0  33 (54.1)  28 (45.9) | 45.0  39 (69.6)  17 (30.4) | 0.084 ^a^ |
| T classification  T_1-2_  T_3-4_ | 13 (21.3)  48 (78.7) | 13 (23.2)  36 (76.8) | 0.805 ^a^ |
| N classification  N_0-1_  N_2-3_ | 30 (49.2)  31 (50.8) | 27 (48.2)  29 (51.8) | 0.917 ^a^ |
| Histological type  WHO type II  WHO type III | 3 ( 4.9)  58 (95.1) | 2 ( 3.6)  54 (96.4) | 1.000 ^b^ |
| Clinical stage  II  III  IVa/b | 2 ( 3.3)  47 (77.0)  12 (19.7) | 3 ( 5.4)  34 (60.7)  16 (33.9) | 0.156 ^b^ |

Note: ^a^, Pearson χ^2^ tests (Asym. Sig., 2-side); ^b^, Pearson χ^2^ tests (Exact. Sig., 2-side)

**Table S3: The treat cycles in the different treatment strategy.**

| **Platinum treatment cycles** | **ICRT** | **CCRT** |
| --- | --- | --- |
| 2 cycles | 58 (92.1) ^a^ | 5 (9.3) |
| 3 cycles | 1 (1.6) | 17 (31.5) |
| ≥4 cycles | 4 (6.3) | 32 (59.3) |
| Average cycle number | 2.22 | 4.38 |

ICRT, IC plus RT;

CCRT, including CCRT or CCRT plus IC;

^a^, case number (percentage).

**Table S4. Sequences of siRNAs and shRNAs invoved in this paper.**

| **Targets** | **siRNAs/shRNAs** | **Direction** | **siRNA/shRNA sequences** |
| --- | --- | --- | --- |
| NC | NC | Sense | r(UUCUCCGAACGUGUCACGU)dTdT |
|  |  | Antisense | r(ACGUGACACGUUCGGAGAA)dTdT |
| BST2 | BST2-si1 | Sense | r(CCUGCAACCACACUGUGAU)dTdT |
|  |  | Antisense | r(AUCACAGUGUGGUUGCAGG)dTdT |
| BST2 | BST2-si2 | Sense | r(GCUCCUGAUCAUCGUGAUU)dTdT |
|  |  | Antisense | r(AAUCACGAUGAUCAGGAGC)dTdT |
| BST2 | BST2-si3 | Sense | r(GCAAUGUCACCCAUCUCCU)dTdT |
|  |  | Antisense | r(AGGAGAUGGGUGACAUUGC)dTdT |
| FGF2 | FGF2-si1 | Sense | r(CCCUCACAUCAAGCUACAA)dTdT |
|  |  | Antisense | r(UUGUAGCUUGAUGUGAGGG)dTdT |
| FGF2 | FGF2-si2 | Sense | r(GGAGUGUGUGCUAACCGUU)dTdT |
|  |  | Antisense | r(AACGGUUAGCACACACUCC)dTdT |
| FGF2 | FGF2-si3 | Sense | r(GGGCAGUAUAAACUUGGAU)dTdT |
|  |  | Antisense | r(AUCCAAGUUUAUACUGCCC)dTdT |
| MVK | MVK-si1 | Sense | r(CCUUGAACUUGAGAACAUU)dTdT |
|  |  | Antisense | r(AAUGUUCUCAAGUUCAAGG)dTdT |
| MVK | MVK-si2 | Sense | r(GCUGUGGCUUUGACUGCUU)dTdT |
|  |  | Antisense | r(AAGCAGUCAAAGCCACAGC)dTdT |
| MVK | MVK-si3 | Sense | r(CCUGCUGACCAACACCAAA)dTdT |
|  |  | Antisense | r(UUUGGUGUUGGUCAGCAGG)dTdT |
| FOXK2 | FOXK2-si1 | Sense | r(CCAUCUGACCUCAAUUUAA)dTdT |
|  |  | Antisense | r(UUAAAUUGAGGUCAGAUGG)dCdA |
| FOXK2 | FOXK2-si2 | Sense | r(CCAGUCAGCCAGUCUUAAU)dTdT |
|  |  | Antisense | r(AUUAAGACUGGCUGACUGG)dAdC |
| FOXK2 | FOXK2-si3 | Sense | r(GGUCAACACCAGCUACCAA)dTdT |
|  |  | Antisense | r(UUGGUAGCUGGUGUUGACC)dTdA |
| BST2 | BST2-sh1 | Sense | CCGGACCTGCAACCACACTGTGATGCTCGAGCATCACAGTGTGGTTGCAGGTTTTTG |
|  |  | Antisense | AATTCAAAAACCTGCAACCACACTGTGATGCTCGAGCATCACAGTGTGGTTGCAGGT |
| BST2 | BST2-sh2 | Sense | CCGGTGCTCCTGATCATCGTGATTCCTCGAGGAATCACGATGATCAGGAGCATTTTTG |
|  |  | Antisense | AATTCAAAAATGCTCCTGATCATCGTGATTCCTCGAGGAATCACGATGATCAGGAGCAT |

NC: negative control.

**Table S5. Sequences of primers invoved in this paper.**

| **Gene** | **Direction** | | | **Primers sequences** | **Vector** |
| --- | --- | --- | --- | --- | --- |
| Cloning primers : | | | | | |
| BST2 | Forward | | 5'-CGGGATCCTAAAGGGGAGATCTGGATGG-3' | | pcDNA3.1(+) |
|  | Reverse | | 5'-GTCCTCGAGATCTCACTGCAGCAGAGCG-3' | |  |
| BST2 wt or ΔGPI | Forward | | 5'- GCTCTAGAATGGCATCTACTTCGTATGA -3' | | pCDH-EF1-MCS-T2A-Puro |
|  | Reverse | | 5'- GCGGATCCCTGCAGCAGAGCGCTGAGGC-3' | |  |
| BST2 Y6,8A | Forward | | 5'- GCTCTAGAATGGCATCTACTTCGGCTGA -3' | | pCDH-EF1-MCS-T2A-Puro |
|  | Reverse | | 5'- GCGGATCCCTGCAGCAGAGCGCTGAGGC-3' | |  |
| Quantitative PCR primers: | | | | | |
| BST2 | | Forward | 5'-TATCTTCACCATCAAGGCCAAC-3' | |  |
|  | | Reverse | 5'-AAGCCATTAGGGCCATCACA-3' | |  |
| FOXK2 | | Forward | 5'-AAAGGCTCGTTCTGGAGGATAG-3' | |  |
|  | | Reverse | 5'-CTGGATGACAGCGAGTTTGG-3' | |  |
| COL17A1 | | Forward | 5'-AGAGGACGGAGTCAAACACG-3' | |  |
|  | | Reverse | 5'-CCTTGAGCAAACGCTTAACAT-3' | |  |
| FGF2 | | Forward | 5'-AAGAGCGACCCTCACATCAAG-3' | |  |
|  | | Reverse | 5'-CGTTTCAGTGCCACATACCAA-3' | |  |
| MVK | | Forward | 5'-AGGTGGACCAAGGAGGATTT-3' | |  |
|  | | Reverse | 5'-CCTGGTATTGCGAGGGACT-3' | |  |
| Bcl-XL | | Forward | 5'-CGTGGAAAGCGTAGACAAGGA-3' | |  |
|  | | Reverse | 5'-AGAGTGAGCCCAGCAGAACC-3' | |  |
| livin | | Forward | 5'-GTCAGTTCCTGCTCCGGTCA-3' | |  |
|  | | Reverse | 5'-GCTGCGTCTTCCGGTTCTT-3' | |  |
| CIAP2 | | Forward | 5'-CTTTTGCTGTGATGGTGGACTC-3' | |  |
|  | | Reverse | 5'-TCTCCTGGGCTGTCTGATGTG-3' | |  |
| Flip | | Forward | 5'-ACCCTCACCTTGTTTCGGACT-3' | |  |
|  | | Reverse | 5'-TGCCTCGGCCCATGTAAT-3' | |  |
| GAPDH | | Forward | 5'-GACTCATGACCACAGTCCATGC-3' | |  |
|  | | Reverse | 5'-AGAGGCAGGGATGATGTTCTG-3' | |  |
