# Supplementary material for: BST2 confers cisplatin resistance via NF-κB signaling in nasopharyngeal cancer
Source: Cell Death Dis. 2017 Jun 15;8(6):e2874–. doi: 10.1038/cddis.2017.271 (PMC5520926; doi:10.1038/cddis.2017.271)
Supplement: Supplementary Figures [file cddis2017271x2.doc]

**Supplemental Figures:**


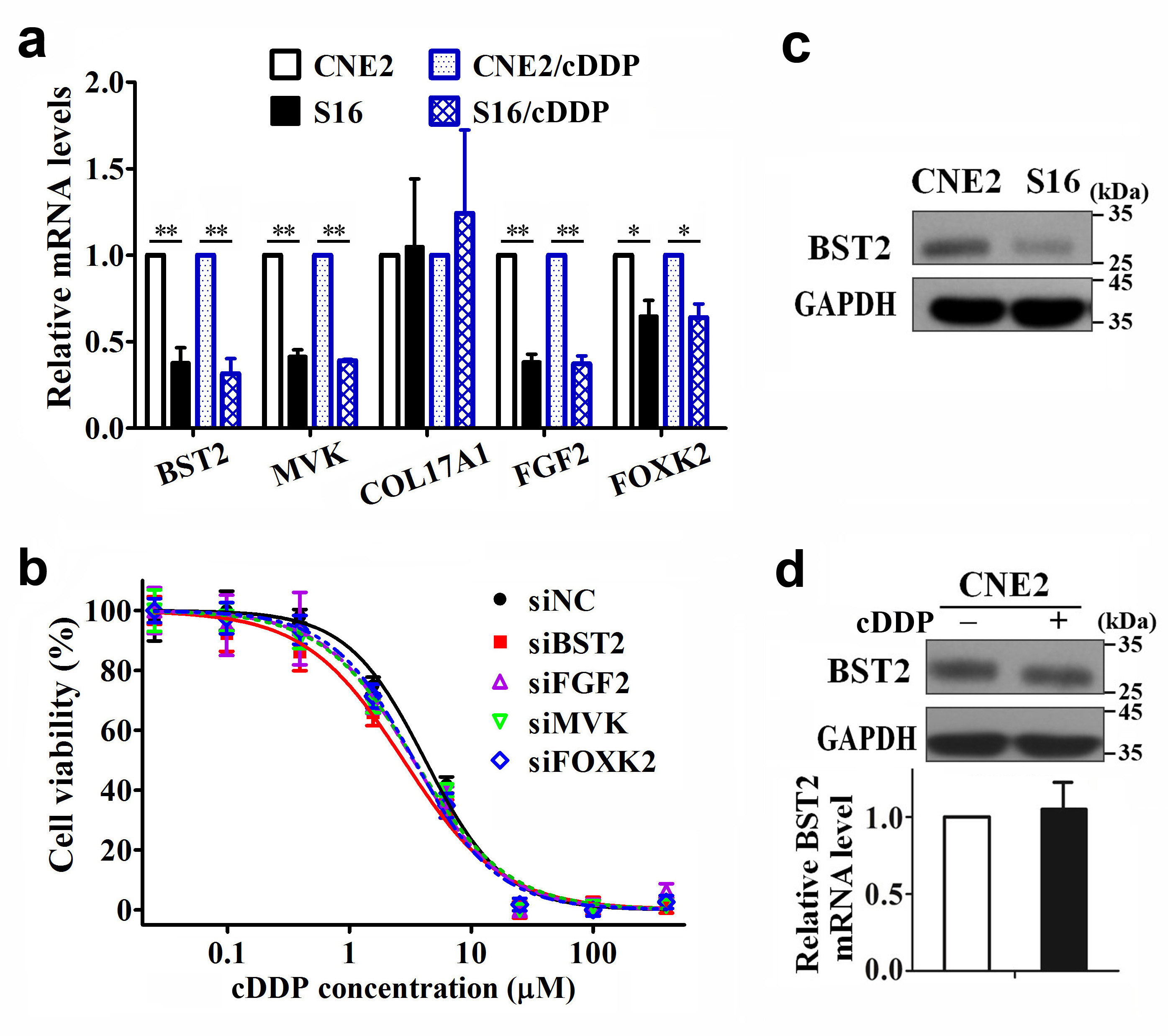


**Figure S1: BST2 is downregulated in cisplatin-sensitive S16 cells compared to the parental CNE2 cells. (a)** Relative mRNA levels of BST2, MVK, Col17A1, FGF2 and FOXK2 in CNE2 and S16 cells treated with or without cisplatin using RT-qPCR assay. **(b)** MTT assays for the effects of candidate genes on cisplatin resistance after knocking down gene expression by transfected with siRNAs (siNC, negative control siRNA; siBST2, BST2 siRNA mixture; siFGF2, FGF2 siRNA mixture; siMVK, MVK siRNA mixture; siFOXK2, FOXK2 siRNA mixture). **(c)** Western blotting (WB) assay for BST2 expression in CNE2 and S16 cells. **(d)** WB (upper) and RT-qPCR (lower) assays for BST2 mRNA and protein levels in CNE2 cells treated with or without cisplatin.

**
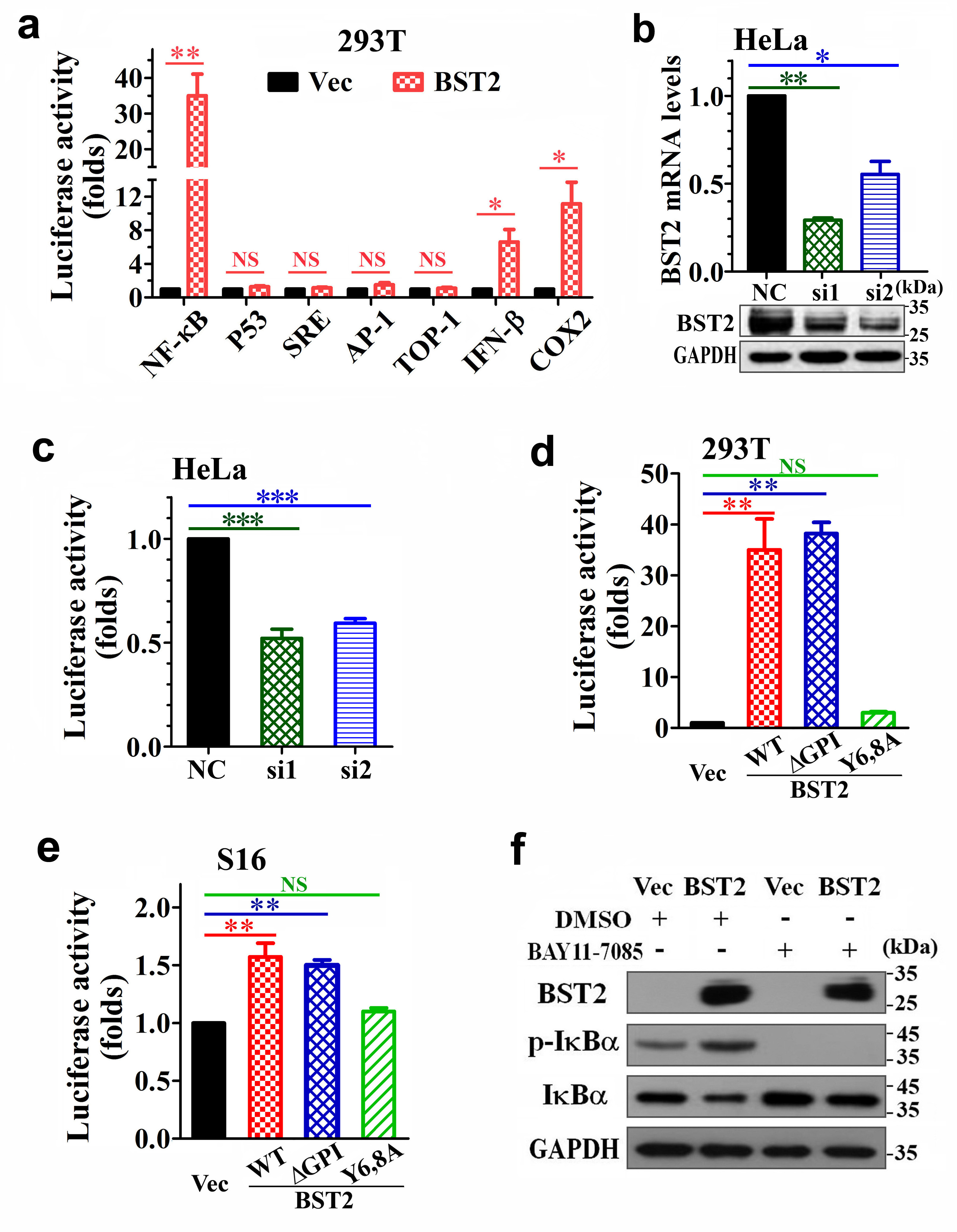
**

**Figure S2: BST2 activates NF-κB pathway.** **(a)** Screening of signaling pathway influenced by BST2 in 293T cells by a luciferase reporter assay as described in *Materials and Methods*. **(b)** BST2 expression level in HeLa cells 24 h after transient transfection with BST2 siRNAs (upper, RT-qPCR assay; lower, WB assay); **(c)** NF-κB luciferase activity in HeLa cells 24 h after BST2 knockdown. **(d, e)** NF-κB luciferase activity in 293T (**d**) or S16 (**e**) cells 24 h after transient transfection of plasmids expressing WT BST2 and BST2 mutants. **(f)** WB assay for key proteins in NF-κB patrhway in S16 cells stably expressing BST2 after 24 h of treatment with 10 μM cisplatin plus 10 μM BAY11-7085 (DMSO was used as a vehicle control). WT, wild-type BST2; ΔGPI and Y6,8A, two types of BST2 mutants; Vec, empty vector; NC, negative control siRNA; si1, si2, BST2 siRNAs. *, p < 0.05; **, p < 0.01; ***, p < 0.001; NS, no significance.


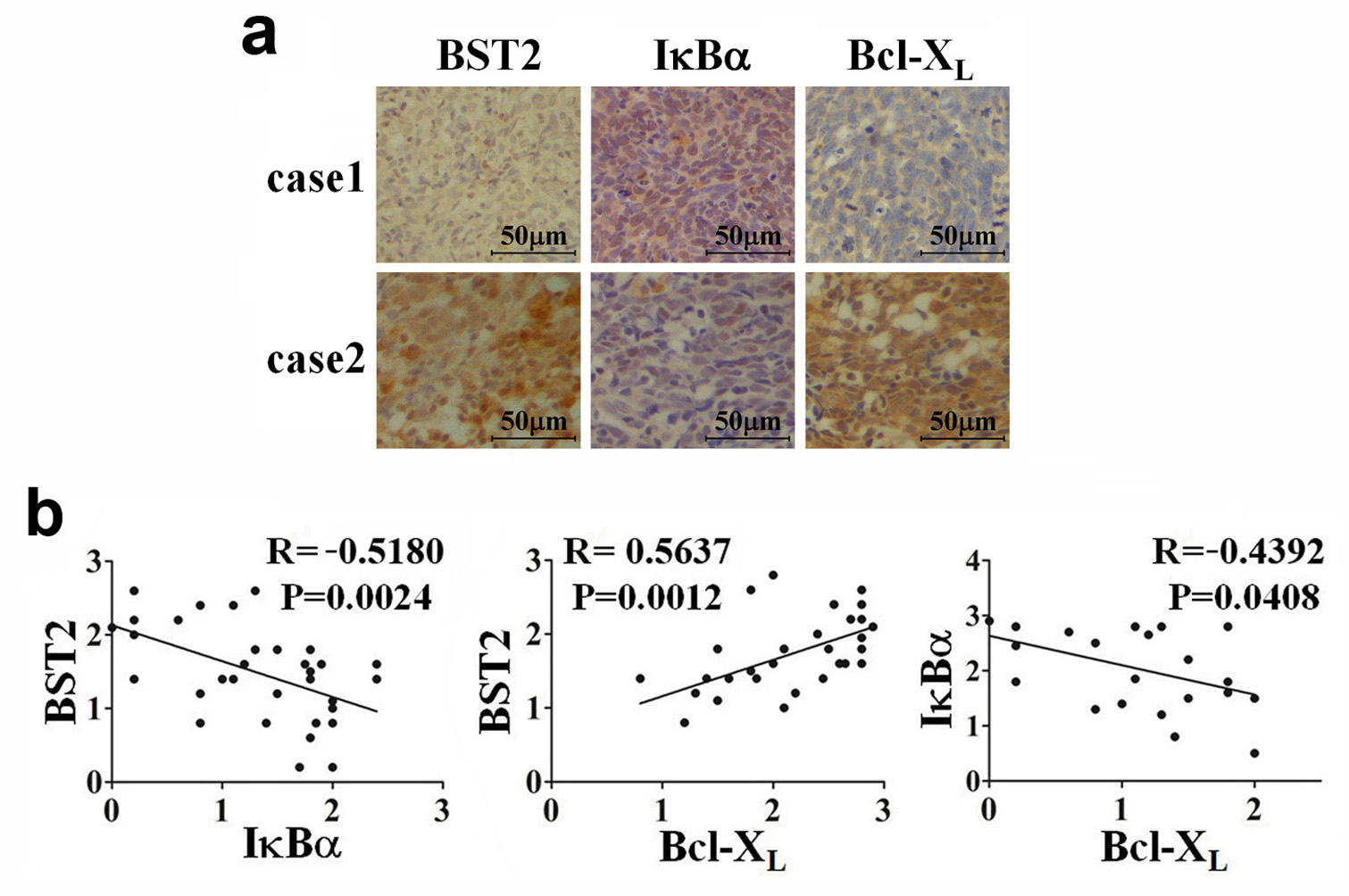


**Figure S3**: **IHC assay assessing the BST2, Bcl-X_L_ and IκBα levels in NPC tissue samples and their relationships. (a)** Reprentative IHC pictures. **(b)** Correlation analysis plots.


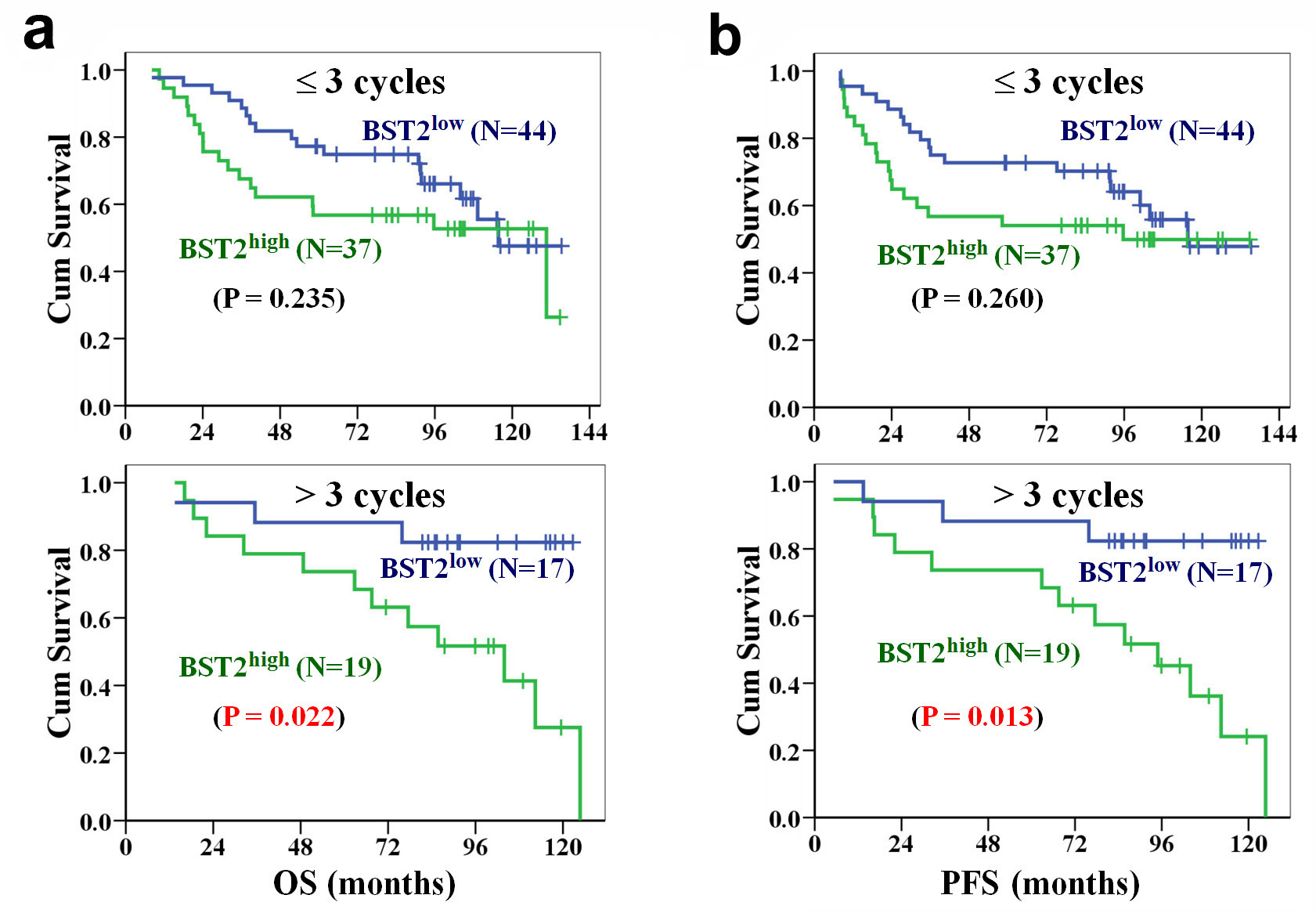


**Figure S4: Stratified survival analysis by cisplatin treatment cycle number.** Patients were divided into 2 groups: treatment cycle number ≤ 3, or >3; and then performed Kaplan-Meier analysis and the log-rank test. **(a)** Overall survival (OS). **(b)** Progression-free survival (PFS). p < 0.05 indicates a significant difference (in red).


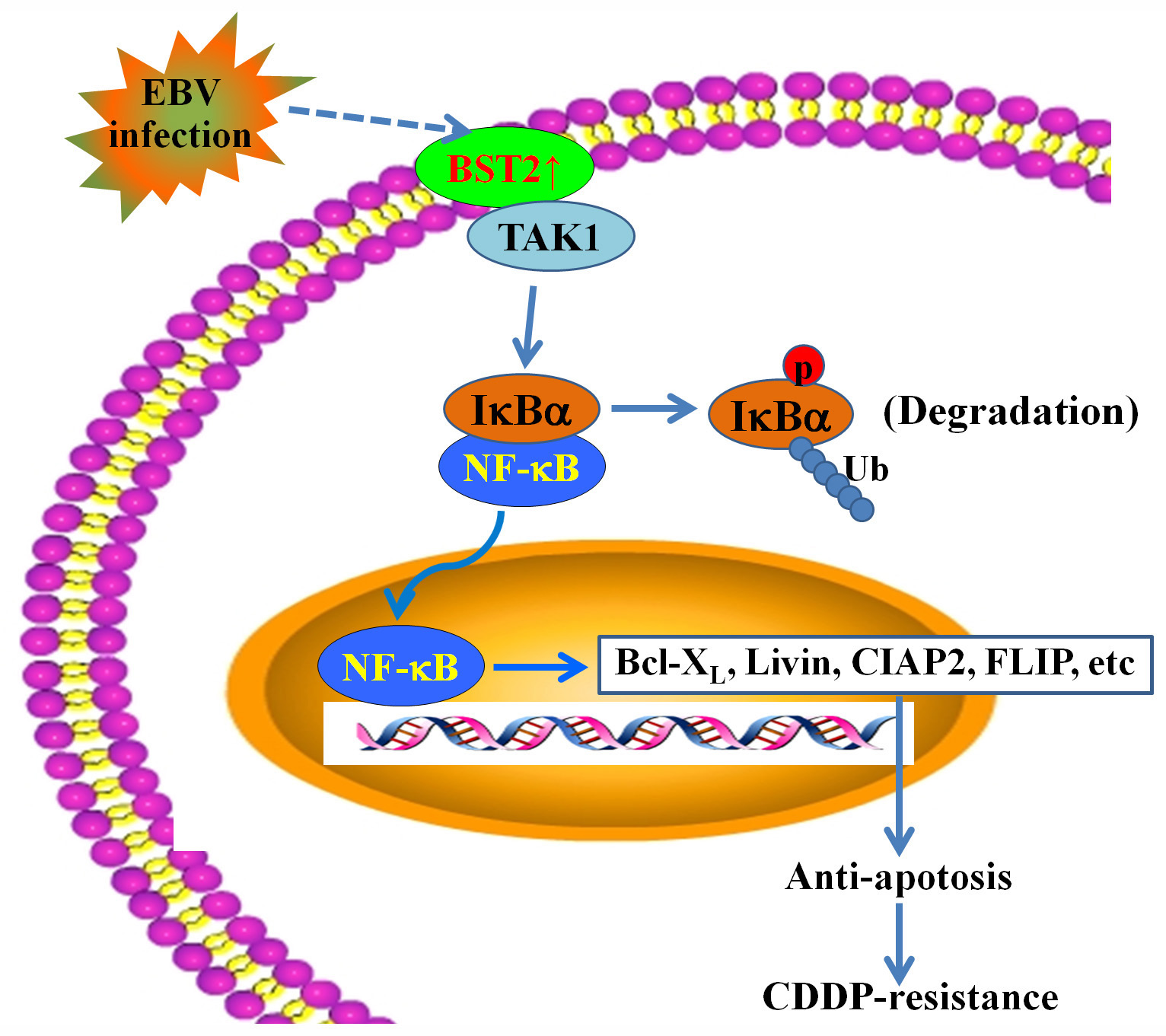


**Figure S5: A brief** mechanism by which BST2 confers cisplatin resistance in nasopharyngeal cancer (NPC). The [ubiquitous](javascript:void(0);) infection of Epstein-barr virus (EBV) always induces the expression of interferon-stimulated genes (ISG), which may be one of reasons mechanisms by which BST2 overexpress in NPC ^39^. The overexpression of BST2 can activate NF-κB signaling through interact with TAK1, consequently triggers the expression of NF-κB-downstream antiapoptotic genes. As a result, cell apoptosis is blocked, and cisplatin resistance is induced in NPC cells.
