# Supplementary figures and images for: BST2 confers cisplatin resistance via NF-κB signaling in nasopharyngeal cancer
Source: Cell Death Dis. 2017 Jun 15;8(6):e2874–. doi: 10.1038/cddis.2017.271 (PMC5520926; doi:10.1038/cddis.2017.271)

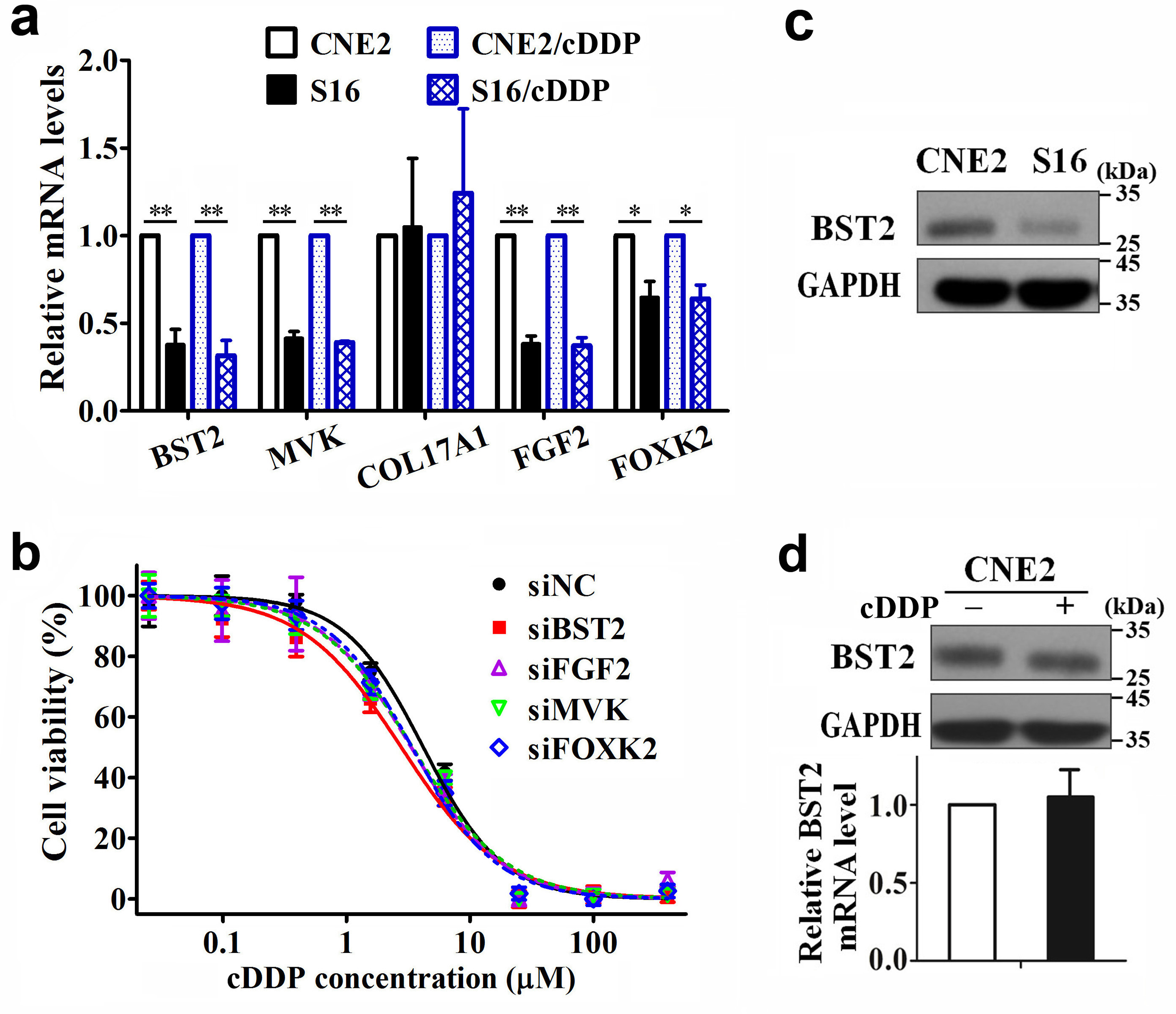

Supplement: Supplementary Figure S1 [file cddis2017271x3.tif]

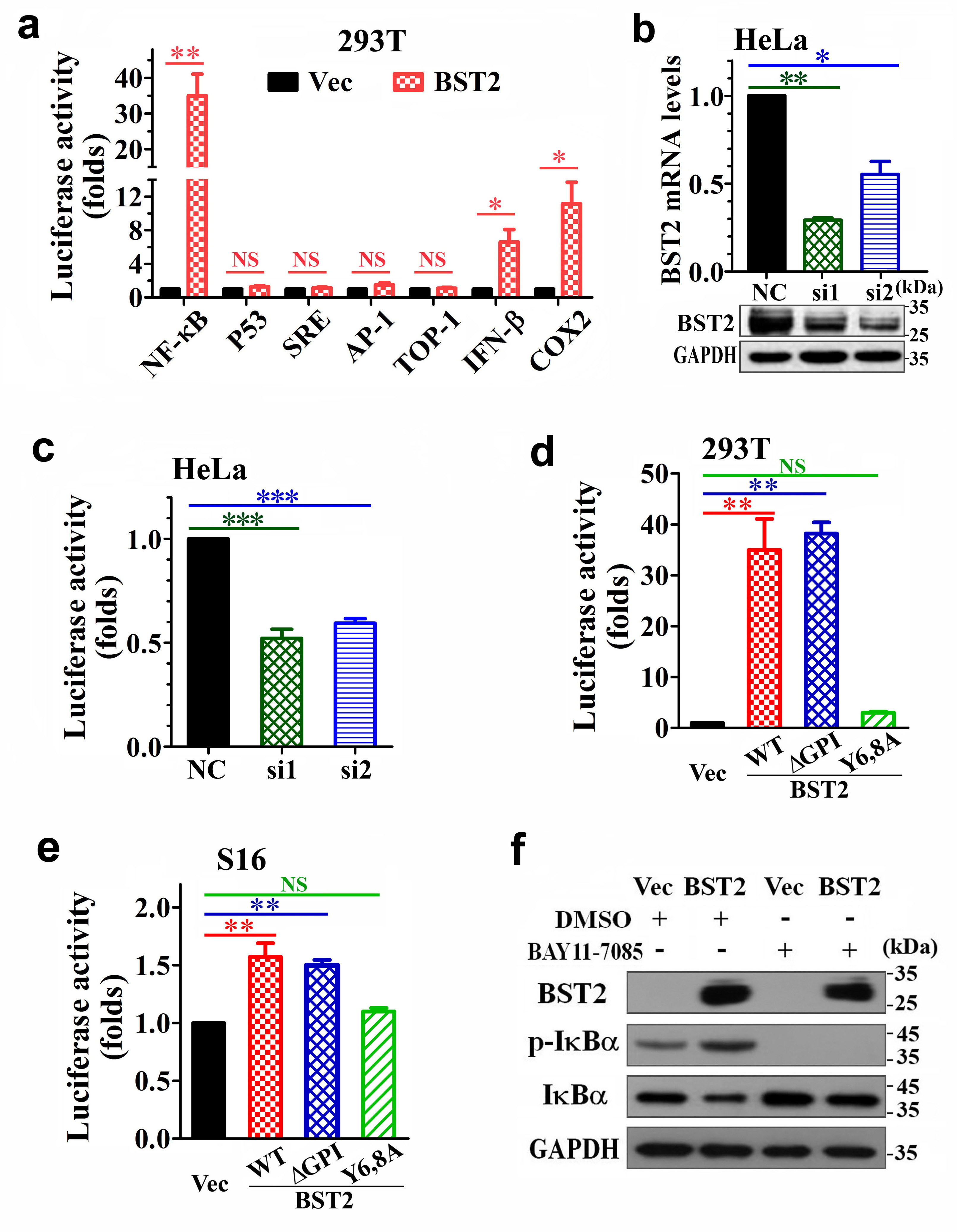

Supplement: Supplementary Figure S2 [file cddis2017271x4.tif]

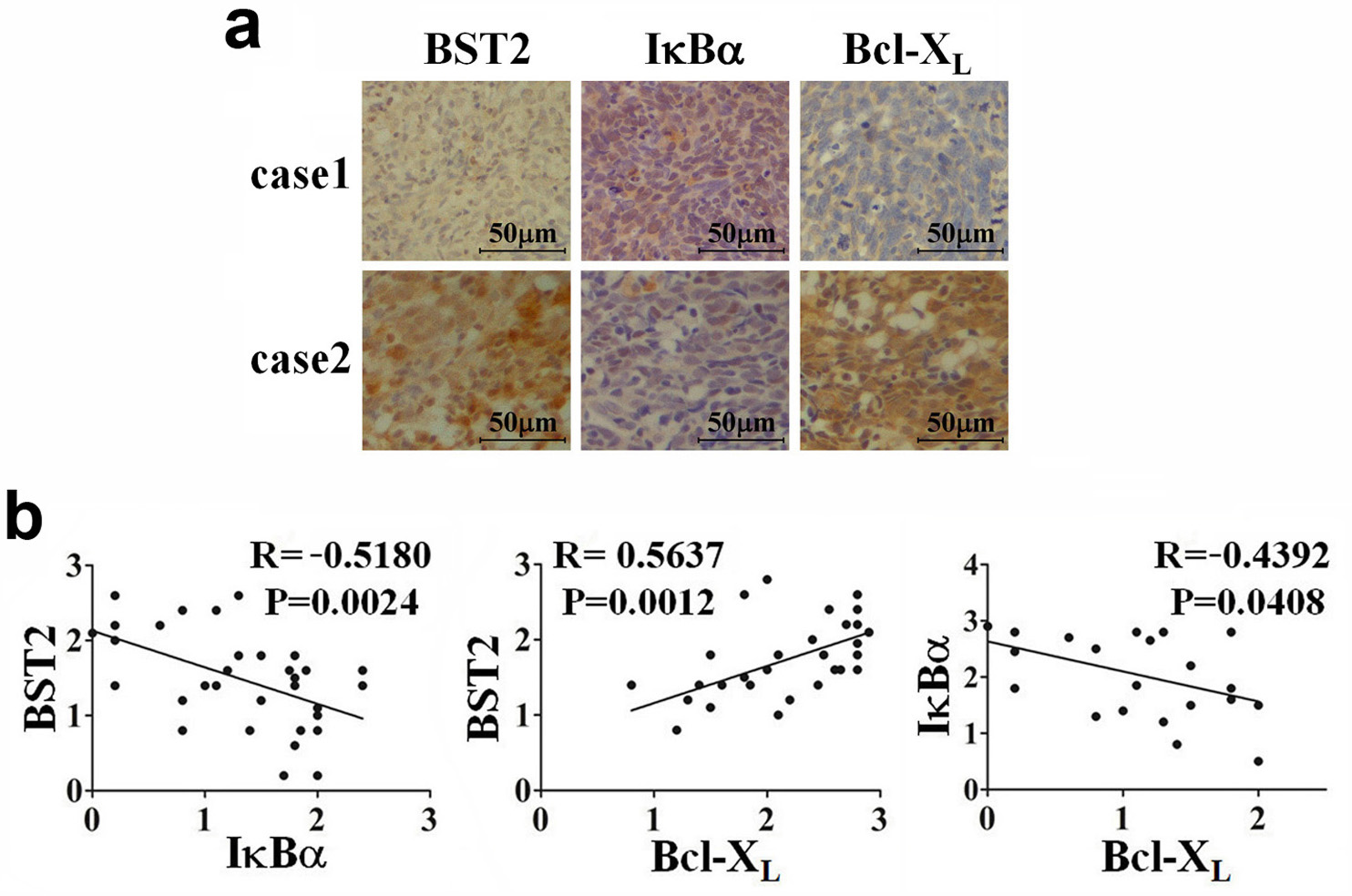

Supplement: Supplementary Figure S3 [file cddis2017271x5.tif]

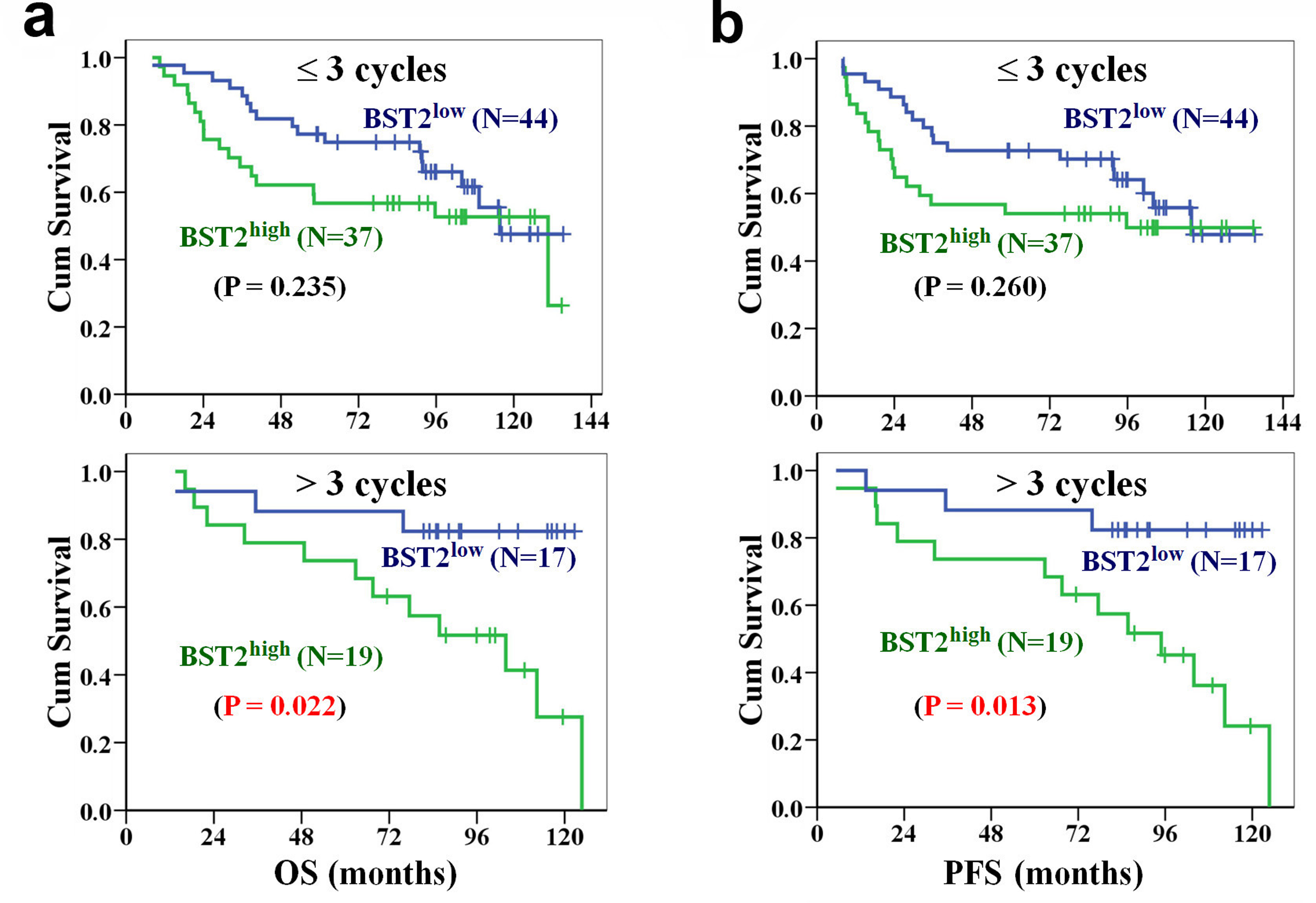

Supplement: Supplementary Figure S4 [file cddis2017271x6.tif]

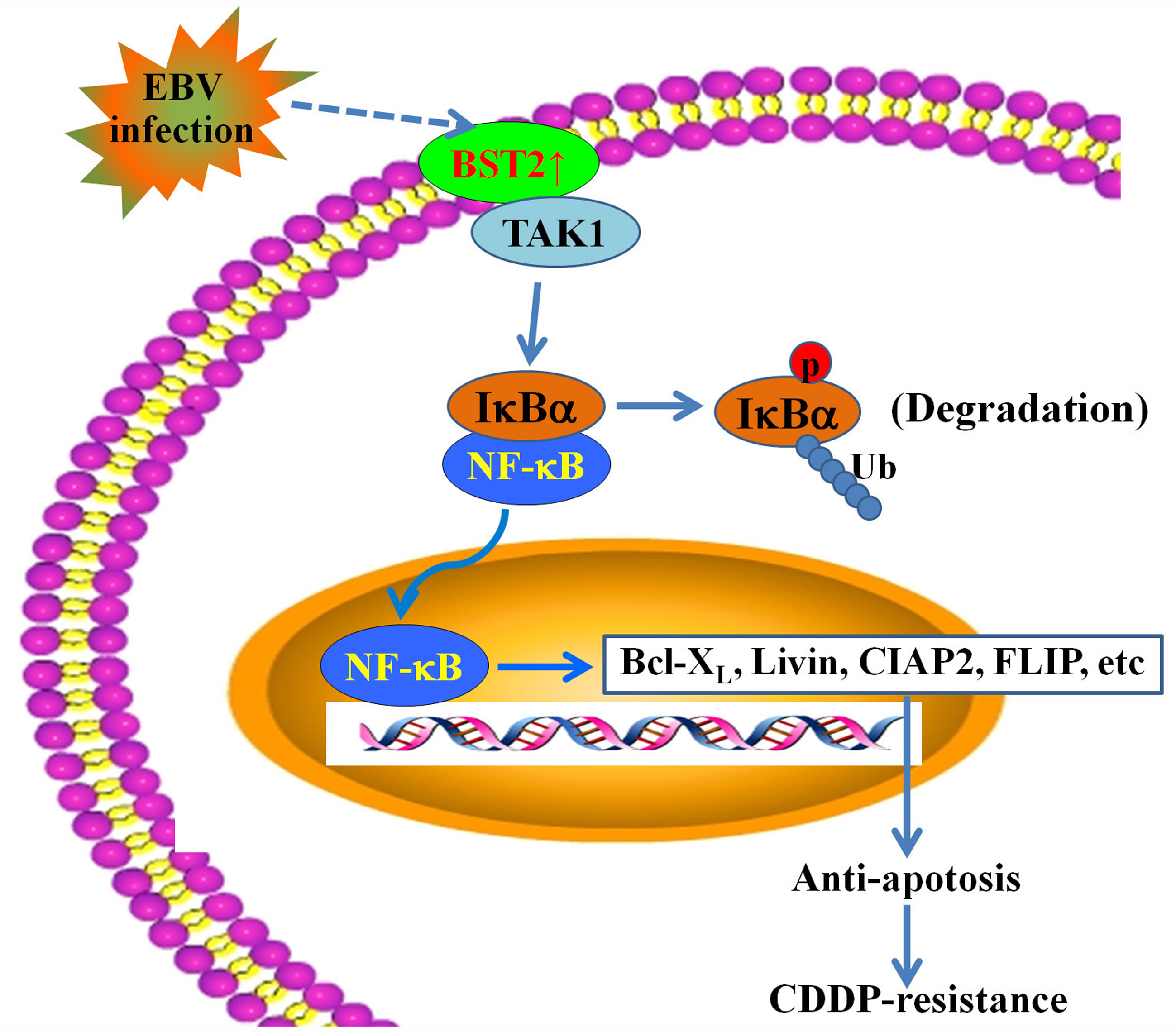

Supplement: Supplementary Figure S5 [file cddis2017271x7.tif]
